# Global Transcriptional Response of Escherichia coli Exposed *In Situ* to Different Low-Dose Ionizing Radiation Sources

**DOI:** 10.1128/msystems.00718-22

**Published:** 2023-02-13

**Authors:** Molly Wintenberg, Lisa Manglass, Nicole E. Martinez, Mark Blenner

**Affiliations:** a Department of Chemical and Biomolecular Engineering, Clemson University, Clemson, South Carolina, USA; b Department of Environmental Engineering and Earth Sciences, Clemson University, Clemson, South Carolina, USA; c Department of Physics and Engineering, Francis Marion University, Florence, South Carolina, USA; d Department of Chemical and Biomolecular Engineering, University of Delaware, Newark, Delaware, USA; University of British Columbia

**Keywords:** RNA-seq, stationary phase, radioactivity, radionuclides, environmental stress

## Abstract

Characterization of biological and chemical responses to ionizing radiation by various organisms is essential for potential applications in bioremediation, alternative modes of detecting nuclear material, and national security. Escherichia coli DH10β is an optimal system to study the microbial response to low-dose ionizing radiation at the transcriptional level because it is a well-characterized model bacterium and its responses to other environmental stressors, including those to higher radiation doses, have been elucidated in prior studies. In this study, RNA sequencing with downstream transcriptomic analysis (RNA-seq) was employed to characterize the global transcriptional response of stationary-phase E. coli subjected to ^239^Pu, ^3^H (tritium), and ^55^Fe, at an approximate absorbed dose rate of 10 mGy day^−1^ for 1 day and 15 days. Differential expression analysis identified significant changes in gene expression of E. coli for both short- and long-term exposures. Radionuclide source exposure induced differential expression in E. coli of genes involved in biosynthesis pathways of nuclear envelope components, amino acids, and siderophores, transport systems such as ABC transporters and type II secretion proteins, and initiation of stress response and regulatory systems of temperature stress, the RpoS regulon, and oxidative stress. These findings provide a basic understanding of the relationship between low-dose exposure and biological effect of a model bacterium that is critical for applications in alternative nuclear material detection and bioremediation.

**IMPORTANCE** Escherichia coli strain DH10β, a well-characterized model bacterium, was subjected to short-term (1-day) and long-term (15-day) exposures to three different *in situ* radiation sources comprised of radionuclides relevant to nuclear activities to induce a measurable and identifiable genetic response. We found E. coli had both common and unique responses to the three exposures studied, suggesting both dose rate- and radionuclide-specific effects. This study is the first to provide insights into the transcriptional response of a microorganism in short- and long-term exposure to continuous low-dose ionizing radiation with multiple *in situ* radionuclide sources and the first to examine microbial transcriptional response in stationary phase. Moreover, this work provides a basis for the development of biosensors and informing more robust dose-response relationships to support ecological risk assessment.

## INTRODUCTION

There has been increasing interest in using biological and chemical responses of various organisms to ionizing radiation for applications in bioremediation, nuclear security, food safety, and crop breeding, with some of this interest motivated by a changing nuclear climate (1, 2). The characterization of the effects of nuclear material on key environmental species has a central role in developing alternative, potentially clandestine, modes of detection using biological systems, informing dose-response relationships to support ecological risk assessment, and in bioremediation of contaminated environments. Realistic exposure scenarios, such as *in situ* exposure to radionuclides relevant to the nuclear fuel cycle, weapons development, and enrichment activities, can provide insight into the response of environmental microorganisms.

The effects of high doses (i.e., grays [Gy]) of ionizing radiation on microorganisms, such as DNA damage (3–5), oxidative stress (6, 7), and apoptosis (5, 7, 8) are well understood. However, the effects of lower doses (i.e., milligrays), particularly low linear energy transfer (LET) radiation, are poorly understood (5, 8–10) as responses and repair pathways are more nuanced. “Low” in the context of this work refers generally to dose and rose rates not anticipated meaningfully impact cellular function. Understanding the biological response of microbial systems subjected to low, environmentally relevant dose rates of ionizing radiation would support a better understanding of the fundamental science underlying new detection, dose-response relationships, and bioremediation applications. Moreover, studying radiation-induced responses at different points within the stationary phase with multiple types of emitters would provide a larger picture of microbial response and be more relevant to the majority population of cells in the environment (11).

Escherichia coli is an ideal system to study the microbial transcriptional response to low-dose ionizing radiation because it is well characterized with an annotated genome (12) and responses to other types of stress, such as nutrient starvation (13, 14), oxidative stress (15, 16), envelope stress (17), and temperature changes (18–20), are known. Previous studies have used E. coli as a model system to characterize the biological effects of high doses of ionizing radiation (21, 22) on DNA damage (23–25), oxidative stress (26), and transcription (27).

This work employs exposure of E. coli to radionuclides relevant to nuclear fuel cycle and enrichment activities *in situ*. This, combined with gene expression analysis, seeks to improve understanding of microbial response to low doses of ionizing radiation. The preferred metrics for quantifying exposure of biota to ionizing radiation are the absorbed dose and absorbed dose rate, where the absorbed dose is the average energy imparted to matter by ionizing radiation, per unit mass, with units of grays (Gy), where 1 Gy is equivalent to 1 J kg^−1^. The target absorbed dose rate of ~10 mGy day^−1^ for this experiment is consistent with the highest Department of Energy dose rate guideline for radiation protection of the environment (9) and is comparable to dose rates observed at some sites with legacy contamination (28). Although the referenced DOE guidelines do not specifically address microorganisms, and our dose rates are approximate, we consider them to be representative of a “low dose rate,” particularly as a recent National Academies report concerning low-dose radiation research defined, in the context of humans, low doses to be below 100 mGy and low dose rates to be less than 5 mGy h^−1^ (29).

The radionuclides considered, namely, ^239^Pu, (half-life [*t*_1/2_] = 24,110 years), tritium (^3^H) (*t*_1/2_ = 12.3 years), and ^55^Fe (*t*_1/2_ = 2.7 years), decay by alpha emission, beta emission, and electron capture, respectively. These radionuclides make interesting comparisons because of the type and energy of radiations emitted as well as being of interest in nuclear sensing applications. ^3^H and ^55^Fe are both common nuclear activation products and ^239^Pu is a fissile nuclide that can be made into a fission weapon or thermonuclear weapon “trigger” (7, 30, 31). Tritium is a pure beta emitter, meaning it has no other associated emissions. ^55^Fe emits electrons and characteristic X rays of similar energy to that of the tritium beta emission. ^239^Pu primarily emits alpha particles, with lesser frequencies of X rays, gamma rays, and electrons (32); note that no radionuclide is a “pure” X-ray or alpha emitter. Of the radionuclides relevant to nuclear activities, these three were chosen because of the similarity in energy emission between ^3^H and ^55^Fe as well as the chemical similarity of iron and plutonium; this along with the different radiations emitted make interesting comparisons when considering *in situ* radionuclides (33).

Ultimately, the goal of this work was to characterize the response of E. coli to low-dose ionizing radiation. To do so, an RNA sequencing approach with downstream transcriptomic analysis (RNA-seq) was used to investigate the global transcriptional response of stationary-phase E. coli DH10β exposed *in situ* to ^239^Pu, ^3^H, or ^55^Fe for 1 day and 15 days. Differential expression analysis revealed significant changes in gene expression of E. coli at the two times studied with the three radionuclide sources. These exposures induced differential expression of E. coli genes involved in biosynthesis pathways of nuclear envelope components, amino acids, and siderophores, transport systems such as ABC transporters and type II secretion proteins, and initiation of stress response and regulatory systems of temperature stress, the RpoS regulon, and oxidative stress. The results and analysis presented here provide new insights into the transcriptional response of a model bacterium to *in situ* low-dose ionizing radiation exposure.

## RESULTS

### Experimental approach to *in situ* exposure.

**FIG 1:**
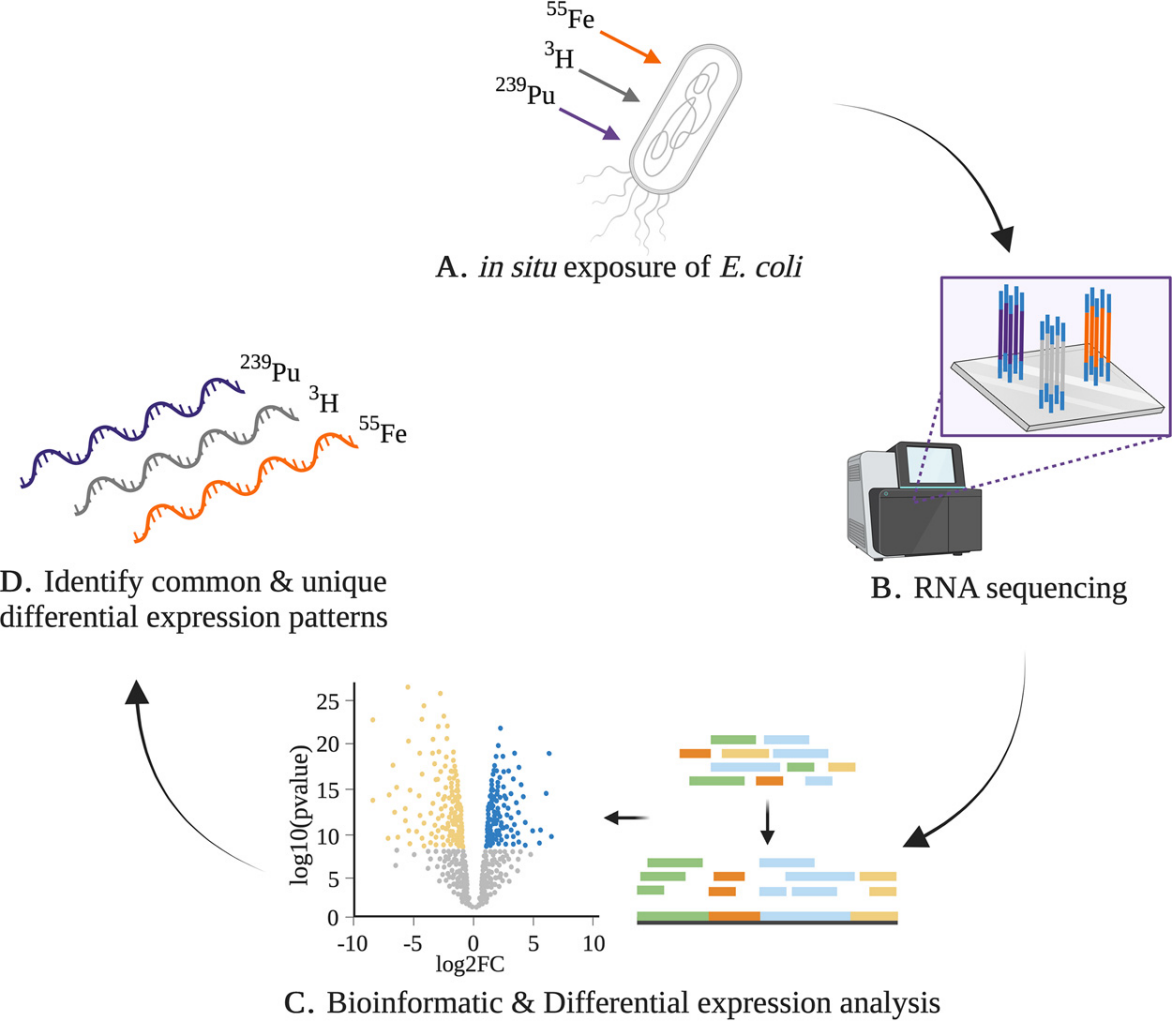
Overview of (A) radiological exposure scenarios of ^239^Pu, ^3^H, and ^55^Fe with stationary-phase *E. coli* using (B) RNA-seq and (C) differential expression analysis to (D) identify unique transcriptional signatures of radiation exposure. The figure was made with BioRender.

To begin characterization of the stationary-phase bacterial response to low-dose ionizing radiation, E. coli DH10β was subjected to continuous exposure to ^239^Pu, ^3^H, or ^55^Fe for 15 days (Fig. 1). Stationary-phase cells were used to represent the majority of cells within a communal environment (11). Growth of E. coli was monitored with optical density measurements at 600 nm (OD_600_) throughout the 15-day study (see Fig. S1A in the supplemental material). Gene expression levels of mRNA isolated from irradiated and nonirradiated E. coli were measured at 1 day and 15 days through RNA sequencing (RNA-seq) and downstream transcriptomic analysis. Illumina sequencing was performed on 30 RNA samples and generated ~508 million reads in total (Table S1). The E. coli K-12 reference genome served as the framework for read alignment and transcript mapping. Gene abundance estimates were used in pairwise comparisons of ^239^Pu-, ^3^H-, or ^55^Fe-treated samples and nonirradiated controls (Fig. S2) to determine differential expression based on a generalized linear model utilizing a negative binomial distribution. Pairwise comparison of ^55^Fe and a stable iron control should provide radiological effects of radioiron and decouple chemical effects by control reference gene expression. Moreover, without a stable analog, nonirradiated E. coli serves as the reference for pairwise comparisons to ^239^Pu. Genes were determined to be differentially expressed if the absolute value of the log_2_ fold change (|log_2_ FC|) was >2 and had an adjusted *P* value of <0.05.

10.1128/msystems.00718-22.1 FIG S1 (A) Visual overview of sample flask inoculation made with Biorender; (B) flask growth of E. coli in M9 minimal medium displayed as optical density at 600 nm over the period of study for ^239^Pu (■), ^3^H (○), ^55^Fe (*), the control (◊), and stable FeCl_3_ (●). Download FIG S1, PDF file, 1.3 MB. Copyright © 2023 Wintenberg et al. 2023 Wintenberg et al. https://creativecommons.org/licenses/by/4.0/ This content is distributed under the terms of the Creative Commons Attribution 4.0 International license.

10.1128/msystems.00718-22.2 FIG S2 Principal-component analysis of all RNA-seq samples in this study. Download FIG S2, PDF file, 0.01 MB. Copyright © 2023 Wintenberg et al. 2023 Wintenberg et al. https://creativecommons.org/licenses/by/4.0/ This content is distributed under the terms of the Creative Commons Attribution 4.0 International license.

10.1128/msystems.00718-22.5 TABLE S1 Characteristics of RNA-seq data in this study. Download Table S1, DOCX file, 0.02 MB. Copyright © 2023 Wintenberg et al. 2023 Wintenberg et al. https://creativecommons.org/licenses/by/4.0/ This content is distributed under the terms of the Creative Commons Attribution 4.0 International license.

### Overlapping and unique global transcriptional responses characterize *in situ* exposure of E. coli.

**FIG 2:**
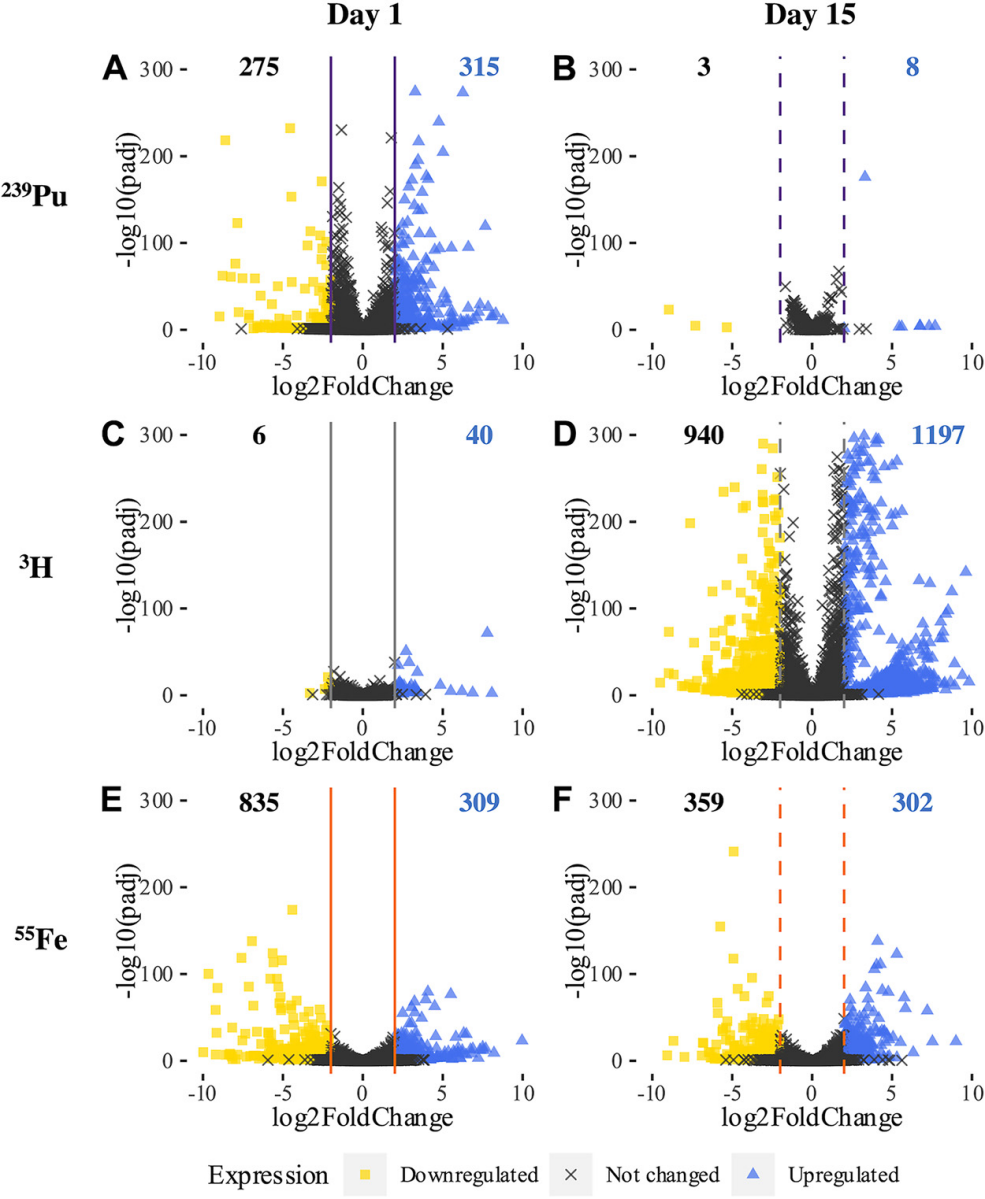
Volcano plots displaying the global transcriptional response of *E. coli* after 1 day (solid line) or 15 days (dashed line) of exposure to (A and B) ^239^Pu, (C and D) ^3^H, or (E and F) ^55^Fe sources are represented by the Benjamin-Hochberg adjusted *P* value (padj) compared to the |log_2_ FC|. Differential expression significance was set with the statistical parameters adjusted *P* value (padj) of <0.05 and |log_2_ FC| > 2, with reference expression baseline set to the nonirradiated control. Genes upregulated, downregulated, and not significantly changed by treatment are represented by blue triangles, yellow squares, and black X’s, respectively.

Significant change in gene expression was induced in E. coli for all radiological exposures considered. The global transcriptional responses of E. coli following 1 day and 15 days of individual radionuclide exposures are shown with volcano plots in Fig. 2, and the numbers of differentially expressed genes are displayed within the plots. Whole-transcriptome analysis with multiple time points in stationary phase demonstrates response dynamics, as the transcriptional response of E. coli to each type of radionuclide varied with time. Exposure to ^239^Pu induced a moderate change in gene expression in E. coli after 1 day but a minimal change after 15 days (Fig. 2A and B), with 590 (13.8% coding DNA sequences [CDSs]) and 11 (0.3% CDSs) differentially expressed genes at 1 day and 15 days, respectively. The opposite response pattern was observed for E. coli with exposure to ^3^H, with extensive differential expression induced after 15 days and a considerably smaller change following 1 day (Fig. 2C and D) with 2,137 (49.9% CDSs) and 46 (1.1% CDSs) differentially expressed genes, respectively. E. coli exposed to ^55^Fe exhibited the largest change in gene expression with respect to a stable iron chloride control after a single day of irradiation and a moderate change following 15 days (Fig. 2E and F). One day of exposure to ^55^Fe induced differential expression of 1,144 (26.7% CDSs) genes in E. coli, whereas 15 days of exposure induced 661 (15.4% CDSs) genes. Unlike with the other two radionuclides studied, the numbers of genes upregulated in E. coli irradiated by ^55^Fe were equivalent after 1 day and 15 days.

**FIG 3:**
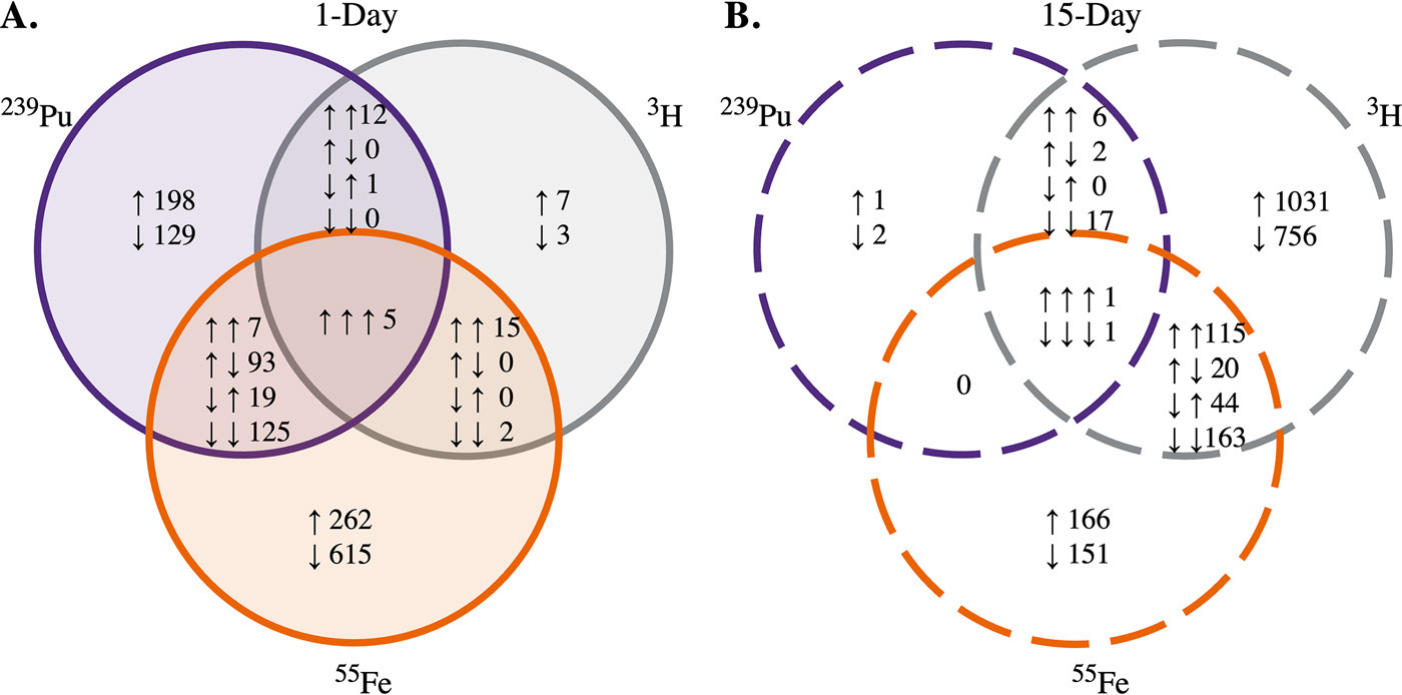
Specific transcriptomic responses of *E. coli* induced by exposure to a single radionuclide source. Results are displayed in the Venn diagrams. The numbers of differentially expressed genes in *E. coli* following (A) 1 day or (B) 15 days of irradiation with ^239^Pu, ^3^H, or ^55^Fe are colored in purple, gray, and orange, respectively. Unions represent genes differentially expressed by more than one radionuclide.

Differentially expressed genes were sorted for uniqueness by the specific radionuclide to which they were exposed and illustrated by Venn diagrams (Fig. 3A and B) to identify potential radionuclide-discriminating responses or commonly induced responses. The numbers of upregulated and downregulated genes (represented by arrows in Fig. 3) in E. coli following exposure to ^239^Pu (purple), ^3^H (gray), or ^55^Fe (orange) for 1 day (Fig. 3A) and 15 days (Fig. 3B) are shown in the Venn diagrams. Differential expression analysis revealed exposure to a single source induced unique expression of several genes after 1 day and 15 days. Moreover, other genes were differentially expressed by more than one radionuclide, as seen in the unions of the Venn diagrams, indicating common responses in E. coli to the absorbed dose rate studied.

### Overrepresentation analysis of differentially expressed genes identified key pathways induced by *in situ* exposure.

**FIG 4:**
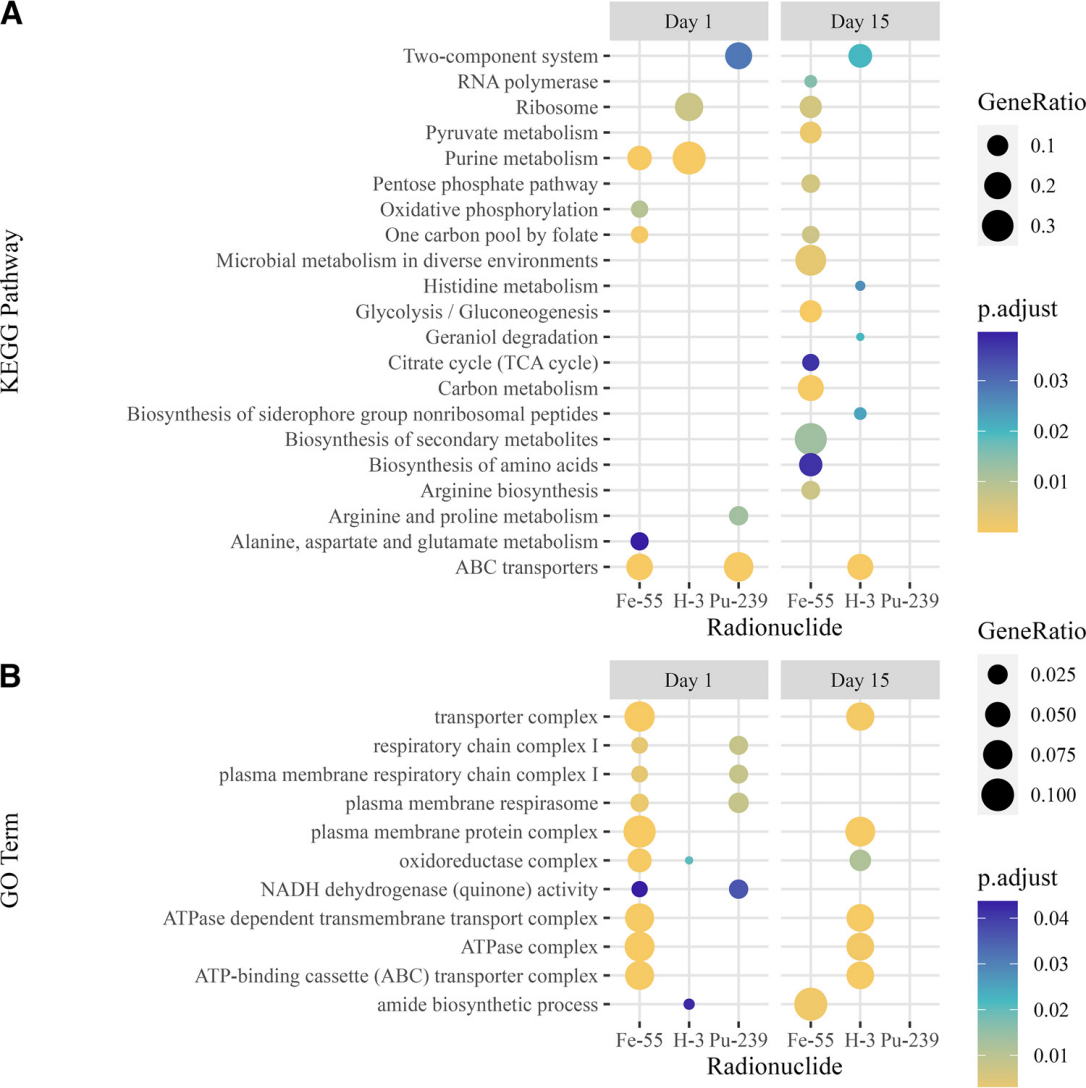
(A) Dot plot displays of KEGG pathways enriched in *E. coli* following 1 day or 15 days of ^55^Fe, ^3^H, or ^239^Pu irradiation identified by overrepresentation analysis; (B) dot plot displays of GO terms significantly overrepresented in *E. coli* following 1 day or 15 days of ^55^Fe, ^3^H, or ^239^Pu irradiation identified by overrepresentation analysis. The color gradient displays the adjusted enrichment *P* value (p.adjust), and the size of the dots denotes the gene ratio of significantly annotated genes to the total number of genes within the pathway.

Functional annotations and enrichment analyses of differential expression results were performed to give biological context to differentially expressed genes. Overrepresentation analysis of differentially expressed genes was performed with clusterProfiler (34, 35) using KEGG pathways (36) for E. coli to identify pathways significantly changed by irradiation. Comparison of enriched pathways by different radionuclide exposures was made with the compareCluster function (34). Results of this analysis indicate key similarities and differences in the response of E. coli to the three different radiological exposures and the two times studied (Fig. 4A). Significant pathways in E. coli induced by irradiation were determined via overrepresentation analysis through calculation of a Benjamini-Hochberg adjusted *P* value and applying a cutoff for significance of <0.05 (Fig. 4A). Several of pathways in E. coli were significantly overrepresented by more than one radionuclide. Purine metabolism (eco00230) was overrepresented following 1 day of either ^55^Fe or ^3^H exposure. ABC transporters (eco02010) were also overrepresented in E. coli after 1 day of either ^55^Fe or ^239^Pu exposure and 15 days of ^3^H exposure. Exposure to ^55^Fe induced several pathways in E. coli related to carbohydrate metabolism, such as glycolysis/gluconeogenesis (eco00010), the citrate cycle (eco00020), and the pentose phosphate pathway (eco00030).

Overrepresentation analysis of differentially expressed genes annotated with GO terms was also performed with clusterProfiler using the enrichGO function for E. coli to identify GO terms significantly changed by irradiation. Key similarities and differences between radionuclides were identified (Fig. 4B). Genes differentially expressed by both 1 day of exposure to ^55^Fe and 15 days of exposure to ^3^H mapped to several overrepresented GO terms for transporter complex, plasma membrane complex, oxidoreductase complex, ATPase-dependent transmembrane complexes, and ABC transporters (Fig. 4B). A single day of exposure to either ^239^Pu or ^55^Fe induced similar changes in gene expression in E. coli with overrepresented GO terms of respiratory chain complex I, NADH dehydrogenase activity, and plasma membrane respirasome respiratory chain complex I (Fig. 4B).

### Common changes in central and energy metabolism were observed for all radionuclide exposures.

KEGG pathway and GO overrepresentation analyses of differentially expressed genes in E. coli demonstrate significant changes in gene expression to carbohydrate and energy metabolism following exposure to ^3^H or ^55^Fe and not ^239^Pu (Table 1). Of the 9 key enzymes in gluconeogenesis, five of them were significantly upregulated in E. coli exposed to ^55^Fe and moderately upregulated (|log_2_ FC| > 1.6) by ^3^H exposure for 15 days. Genes encoding enzymes in the citric acid cycle (tricarboxylic acid [TCA]) were significantly downregulated after 1 day of ^55^Fe exposure but upregulated by 15 days of ^3^H exposure. Similar expression patterns were observed with components in the pentose phosphate pathway, where key genes, such as those encoding transketolase 2, transaldolase A, phosphopentomutase, and fructose-bisphosphate aldolase class II, were upregulated by 15 days of ^3^H or ^55^Fe exposure and downregulated by 1 day of ^55^Fe exposure.

**TABLE 1:** Select differentially expressed genes involved in carbohydrate and energy metabolism in E. coli following 1 day and 15 days of exposure to a ^239^Pu, ^3^H, or ^55^Fe source

Gene ID	Product name	Fold change under radionuclide^*a*^					
		^239^Pu		^3^H		^55^Fe	
		Day 1	Day 15	Day 1	Day 15	Day 1	Day 15
Gluconeogenesis							
*pgi*	Glucose-6-phosphate isomerase				1.87		2.29
*fbp*	Fructose-1,6-bisphosphatase 1		−0.19		1.77		2.33
*fbaB*	Fructose-bisphosphate aldolase class I	2.16	0.43	−1.04	1.55	−4.43	
*fbaA*	Fructose-bisphosphate aldolase class II	−0.85			2.38		2.54
*gapA*	Glyceraldehyde-3-phosphate dehydrogenase A	1.09		1.33	−0.78	2.39	2.93
*pgk*	Phosphoglycerate kinase	−0.84			3.05		1.45
*gpmA*	2,3-Bisphosphoglycerate-dependent phosphoglycerate mutase	−0.92		−0.73	3.15		0.88
*gpmM*	2,3-Bisphosphoglycerate-independent phosphoglycerate mutase	−1.00			1.80	2.37	1.64
*eno*	Enolase	−0.54			1.36		2.43
TCA cycle							
*frdA*	Fumarate reductase flavoprotein subunit	−2.40			4.43	−2.49	
*frdB*	Fumarate reductase iron-sulfur protein				4.48	−3.13	
*mdh*	Malate dehydrogenase	2.06			−0.43	−2.32	1.25
*mqo*	Malate:quinone oxidoreductase	2.55	−0.24		0.51	−2.60	
Pentose phosphate pathway							
*tktB*	Transketolase 2	1.07	0.75	−1.39	3.83	−3.91	3.50
*tktA*	Transketolase 1		−0.38		−0.47	2.51	2.02
*talB*	Transaldolase B	0.48			2.83	1.44	3.60
*talA*	Transaldolase A	2.38	0.71	−0.90	3.48	−4.14	3.12
*fbaB*	Fructose-bisphosphate aldolase class I	2.16	0.43	−1.04	1.55	−4.43	
Oxidative phosphorylation							
*nuoA*	NADH:quinone oxidoreductase subunit A	2.70			−2.42		
*nuoE*	NADH:quinone oxidoreductase subunit E	−2.47			5.62	−2.77	
*sdhA*	Succinate:quinone oxidoreductase, FAD binding protein	−4.13			7.96	−5.24	
*atpA*	ATP synthase F1 complex subunit alpha		−1.01	0.80	−2.20		3.20
Nitrogen and sulfur metabolism							
*narG*	Nitrate reductase A subunit alpha	−2.10		2.08	4.68		
*napA*	Periplasmic nitrate reductase subunit NapA	−2.38			3.25	−3.84	
*gltB*	Glutamate synthase subunit GltB		−0.76		−2.37	2.85	

a Absence of a number represents an adjusted *P* value of >0.05.

A similar pattern of changes in expression holds for thiamine metabolism, with downregulation of genes by 1 day of ^239^Pu or ^55^Fe exposure in E. coli and upregulation with 15 days of ^3^H exposure. Genes involved oxidative phosphorylation were also differentially expressed in E. coli following exposure to 9 mGy day^−1^ of ^239^Pu, ^3^H, or ^55^Fe exposure. NADH quinone oxidoreductase subunits, succinate quinone oxidoreductases, and ATP synthase F_1_ complex subunits were differentially expressed in irradiated E. coli (Table 1). Nitrogen and sulfur metabolism genes were similarly differentially expressed in irradiated E. coli. Nitrate reductase subunits and glutamate synthase subunits were highly upregulated by 15 days of ^3^H exposure and downregulated in E. coli by 1 day of ^239^Pu or ^55^Fe exposure as displayed in Table 1.

Differential expression and pathway analyses also revealed changes in expression of genes involved in amino acid biosynthesis and degradation pathways (Table 2). Several genes involved in valine, leucine, and isoleucine degradation (eco00280) and others involved in lysine degradation (eco00310) were significantly upregulated by 15 days of ^3^H exposure. Histidine metabolism (eco00340) genes were also upregulated in E. coli by 15 days of ^3^H exposure and downregulated by 1 day of ^239^Pu or ^55^Fe exposure.

**TABLE 2:** Select differentially expressed genes involved in amino acid metabolism in E. coli following 1 day and 15 days of exposure to ^239^Pu, ^3^H, or ^55^Fe

Gene ID	Product name	Fold change under indicated radionuclide^*a*^					
		^239^Pu		^3^H		^55^Fe	
		Day 1	Day 15	Day 1	Day 15	Day 1	Day 15
*paaF*	2,3-Dehydroadipyl-CoA hydratase			4.18			
*fadB*	Multifunctional enoyl-CoA hydratase	1.25	0.29		3.89	−1.44	
*fadJ*	3-Hydroxyacyl-CoA dehydrogenase FadJ	2.22	0.58		2.34		
*fadI*	3-Ketoacyl-CoA thiolase FadI	1.17	0.49	1.42	3.88	−1.04	
*fadA*	3-Ketoacyl-CoA thiolase	0.82		0.08	2.77	−0.66	0.96
*scpA*	Methylmalonyl-CoA mutase	−3.61			3.55	−1.05	1.81
*ldcC*	Lysine decarboxylase 2	0.87		−2.79			2.19
*cadA*	Lysine decarboxylase 1						−3.47
*patA*	Putrescine aminotransferase		0.69				6.47
*hisI*	Bifunctional phosphoribosyl-AMP cyclohydrolase	−3.21			5.36	−3.43	
*hisA*	Isomerase	−2.74			4.44	−2.07	
*hisH*	Imidazole glycerol phosphate synthase subunit HisH	−8.97	6.75		6.61		
*hisF*	Imidazole glycerol phosphate synthase subunit HisF	−2.50			4.49	−3.29	
*hisB*	Imidazoleglycerol-phosphate dehydratase	−0.75	3.38		2.10		3.49
*hisC*	Histidinol-phosphate aminotransferase	−2.11			4.85	−2.08	
*hisD*	Histidinol dehydrogenase	−2.51	-		6.11	−2.29	-

a Absence of a number represents an adjusted *P* value of >0.05.

Genes in biosynthesis pathways of the nuclear envelope components such as lipopolysaccharide, O-antigen nucleotide sugars, and peptidoglycan were differentially expressed in E. coli following exposure to ^3^H or ^55^Fe (Fig. S3). Similarly, as observed with other processes, genes within these pathways were upregulated by ^3^H after 15 days of exposure and downregulated by 1 day of exposure to ^55^Fe.

10.1128/msystems.00718-22.3 FIG S3 Differentially expressed genes encoding components of the nuclear envelope are displayed within the heat map. Upregulated and downregulated genes are represented by blue and yellow, respectively. Download FIG S3, PDF file, 0.2 MB. Copyright © 2023 Wintenberg et al. 2023 Wintenberg et al. https://creativecommons.org/licenses/by/4.0/ This content is distributed under the terms of the Creative Commons Attribution 4.0 International license.

### Radionuclide exposure modified transport, signaling, and regulatory systems in E. coli.

**FIG 5:**
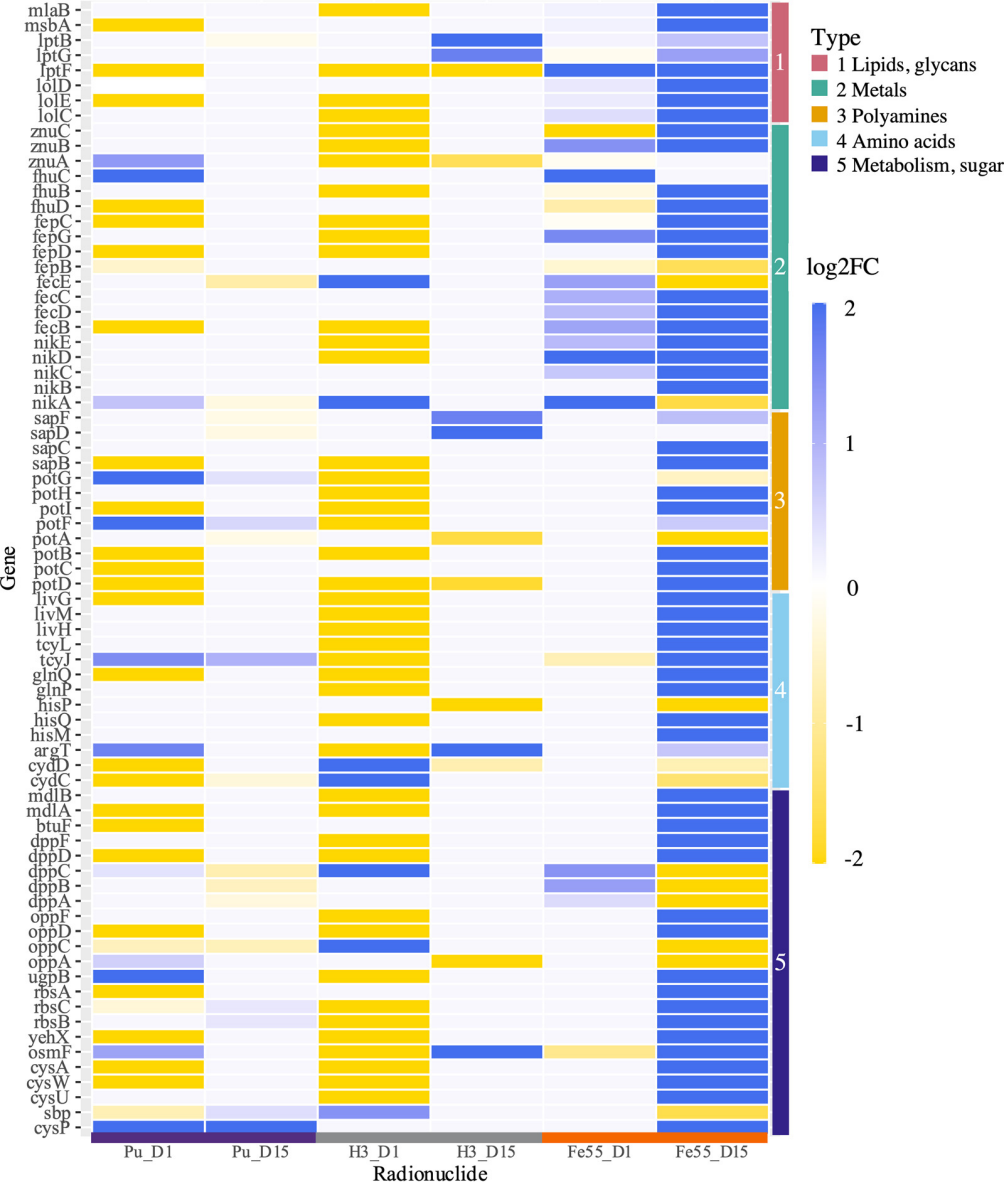
Heat map displaying differential expression of genes involved in various ABC transport systems as denoted by the vertical color bar. The |log_2_ FC| values of upregulated and downregulated genes are shown in blue and yellow, respectively.

Bacterial transport and regulatory systems were found to be significantly changed in irradiated E. coli by overrepresentation analysis (Fig. 4). Several genes encoding components of these systems, including ABC transporters, two-component system (TCS), and the phosphotransferase system (PTS), were differentially expressed. Significant changes in expression of genes involved in ABC transport systems for lipid, amino acid, polyamine, peptide, metal ion, and sugar ABC transporters were found in irradiated E. coli compared to nonirradiated E. coli (Fig. 5). ABC transporter genes were mostly downregulated in E. coli after exposure to ^239^Pu or ^55^Fe for 1 day but were upregulated after 15 days of exposure to ^3^H and are displayed within the heat map. Polyamine transporter genes for putrescine and spermidine were differentially expressed by E. coli exposed to ^3^H or ^55^Fe. Metal ion and complex transporters for nickel (*nikABCDE*), iron III (*fhuBCD*), ferric citrate (*fecBCDE*), and zinc (*znuABC*), were all upregulated after 15 days exposure to ^3^H but downregulated in E. coli by 1 day of ^239^Pu or ^55^Fe exposure.

Several genes encoding components of other bacterial transport, stimulus, and communication systems of PTS and TCS were differentially expressed (Fig. S4). Phosphotransferase genes encoding phosphotransferase enzymes for fructose (*fruABK*), mannose (*manXYZ*), and glucose (*ptsG*), were downregulated in E. coli by 1 day of exposure to ^55^Fe and upregulated by 15 days of exposure to ^3^H (Fig. S4). Genes that have roles in the TCS, such as sensor histidine kinases (*envZ*, *baeS*, *kdpD*, *zraS*, and *cpxA*), the corresponding response regulators (*baeR*, *zraR*, and *cpxR*), the DNA-binding transcriptional dual regulator (*ompR*), and outer membrane porins (*ompCF*), were upregulated by ^3^H after 15 days of exposure and downregulated by 1 day of exposure to ^239^Pu or ^55^Fe (Fig. S4). The opposite expression was found for *phoQ*, representing sensor histidine kinases.

10.1128/msystems.00718-22.4 FIG S4 Differentially expressed genes encoding components of PTS and TCS are displayed within the heat map. Upregulated and downregulated genes are represented by blue and yellow, respectively. Download FIG S4, PDF file, 0.2 MB. Copyright © 2023 Wintenberg et al. 2023 Wintenberg et al. https://creativecommons.org/licenses/by/4.0/ This content is distributed under the terms of the Creative Commons Attribution 4.0 International license.

Siderophore biosynthesis, more specifically enterobactin biosynthesis, was induced in E. coli by 1 day of ^239^Pu exposure (Table 3). Enterobactin biosynthesis consists of 7 genes in E. coli K-12, and following 1 day of exposure to ^239^Pu, 6 genes were upregulated (|log_2_ FC| > 1.7) and one, *entF*, was downregulated in E. coli. Long-term exposure to ^3^H caused the opposite response. As expected in the presence of iron, exposure to a ^55^Fe source did not result in significant changes in gene expression compared to a stable iron control.

**TABLE 3:** Enterobactin genes were differentially expressed in E. coli after 1 day and 15 days of exposure to ^239^Pu or ^3^H

GeneID	Product name	Fold change under indicated radionuclide source^*a*^					
		^239^Pu		^3^H		^55^Fe	
		Day 1	Day 15	Day 1	Day 15	Day 1	Day 15
*entC*	Isochorismate synthase EntC	2.41			−3.09		−0.99
*entB*	Enterobactin synthase component B	1.97	−0.82		−3.24		4.48
*entA*	2,3-Dihydro-2,3-dihydroxybenzoate dehydrogenase	1.97	−1.01	0.70	−2.45		0.70
*entD*	Phosphopantetheinyl transferase EntD	3.15	−0.48		−5.79	−1.35	
*entE*	2,3-Dihydroxybenzoate-AMP ligase	1.70	−0.48		−2.42		0.94
*entF*	Apo-serine activating enzyme	−2.77			5.12	−3.20	
*entH*	Proofreading thioesterase	1.91	−1.17	0.76	−3.40		

a Absence of a number represents an adjusted *P* value of >0.05.

### *In situ* exposure initiated stress responses in irradiated E. coli.

**FIG 6:**
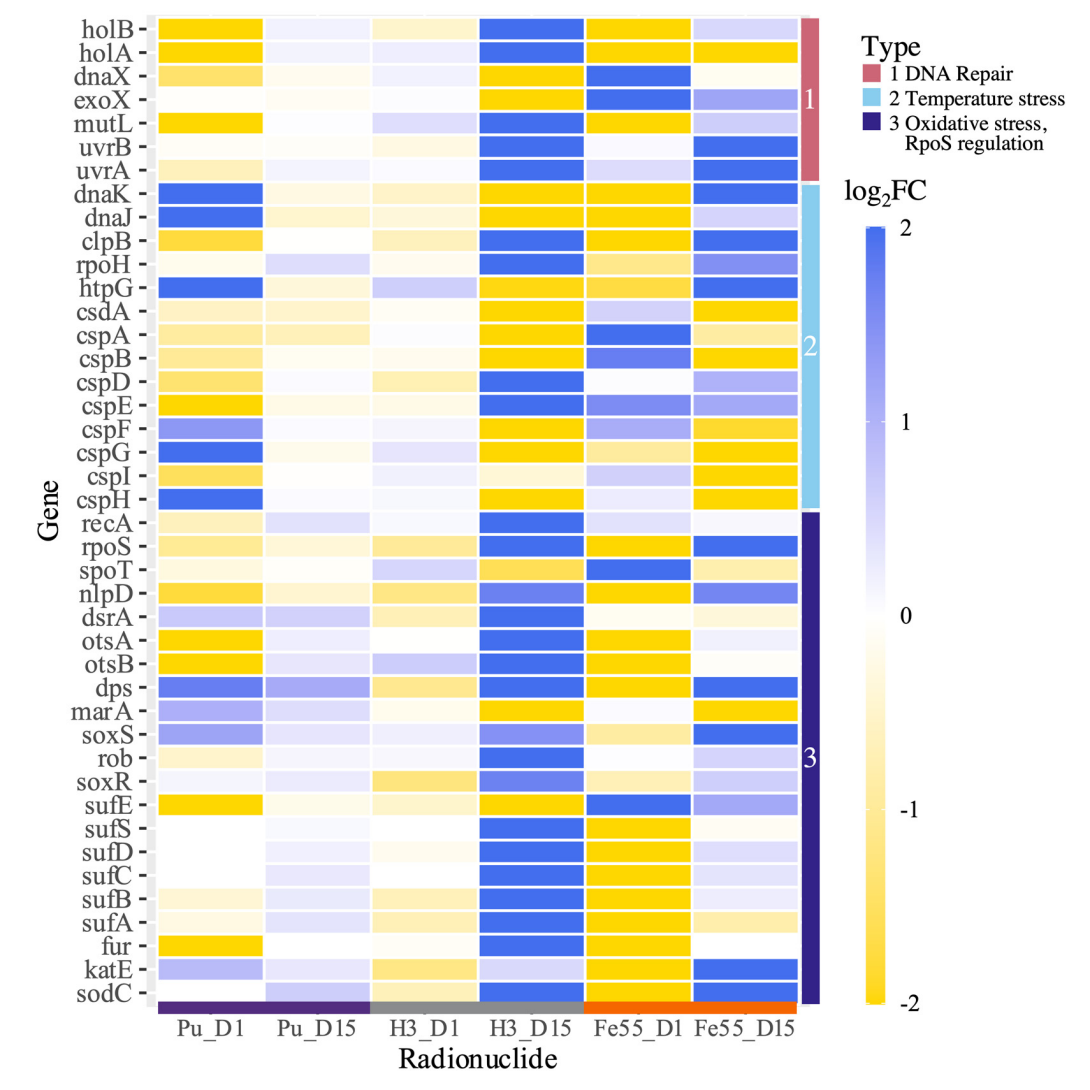
Heat map displaying select differentially expressed genes in *E. coli* exposed to 1 day and 15 days of ^239^Pu, ^3^H, or ^55^Fe that are components of the stress response denoted by the vertical color bar types. The |log_2_ FC| values of upregulated and downregulated genes are shown in blue and yellow, respectively.

Differential expression analysis revealed the expression of genes with key roles in combating general, oxidative, osmotic, and temperature stress in addition to others involved in DNA repair mechanisms in E. coli were significantly changed by exposure to the sources studied (Fig. 6). The heat map displays downregulation of two oxidative stress genes, *sodC* and *katE*, in response to 1 day of ^55^Fe exposure but subsequent upregulation of the genes following 15 days of exposure to ^55^Fe and ^3^H. Other markers of oxidative stress positively regulated by OxyR, including iron-sulfur cluster assembly proteins, DNA-binding transcriptional dual regulator (encoded by *fur*), and an iron-binding and storage protein (encoded by *dps*), were significantly downregulated after 1 day of exposure to ^55^Fe but upregulated after 15 days of ^3^H exposure. Several genes (*rpoS*, *spoT*, *nlpD*, *dsrA*, *otsAB*, and *dts*) controlled by the sigma factor RpoS that play a role in the global and osmotic stress responses were differentially expressed following exposure to ^55^Fe and ^3^H (Fig. 6).

Indicators of cold and heat shock were also expressed. The cold shock protein genes *cspH* and *cspG* were upregulated by 1 day of exposure to ^239^Pu and downregulated by 15 days of ^55^Fe or ^3^H (Fig. 6). Other cold shock protein genes (*cspB*, *cspF*, and *cspG*) were downregulated in E. coli by both 1 day and 15 days of exposure to ^3^H. Key genes involved in heat shock response, including the high-temperature protein G gene *htpG* and heat shock protein chaperone genes *dnaKJ* (37), were significantly upregulated following exposure to 1 day of ^239^Pu and 15 days of ^55^Fe. Differential expression analysis provided some suggestion of DNA strand break repair with significant changes in genes that play a role in repair pathways (Fig. 6). These genes, consisting of genes coding for excision nuclease subunits A and B (*uvrA* and *uvrB*, respectively), DNA mismatch repair protein (*mutL*), exonuclease X (*exoX*), and three subunits of DNA polymerase III (*dnaX*, *holA*, and *holB*), were differentially expressed.

### Unique responses to a single radionuclide can discriminate between sources.

**FIG 7:**
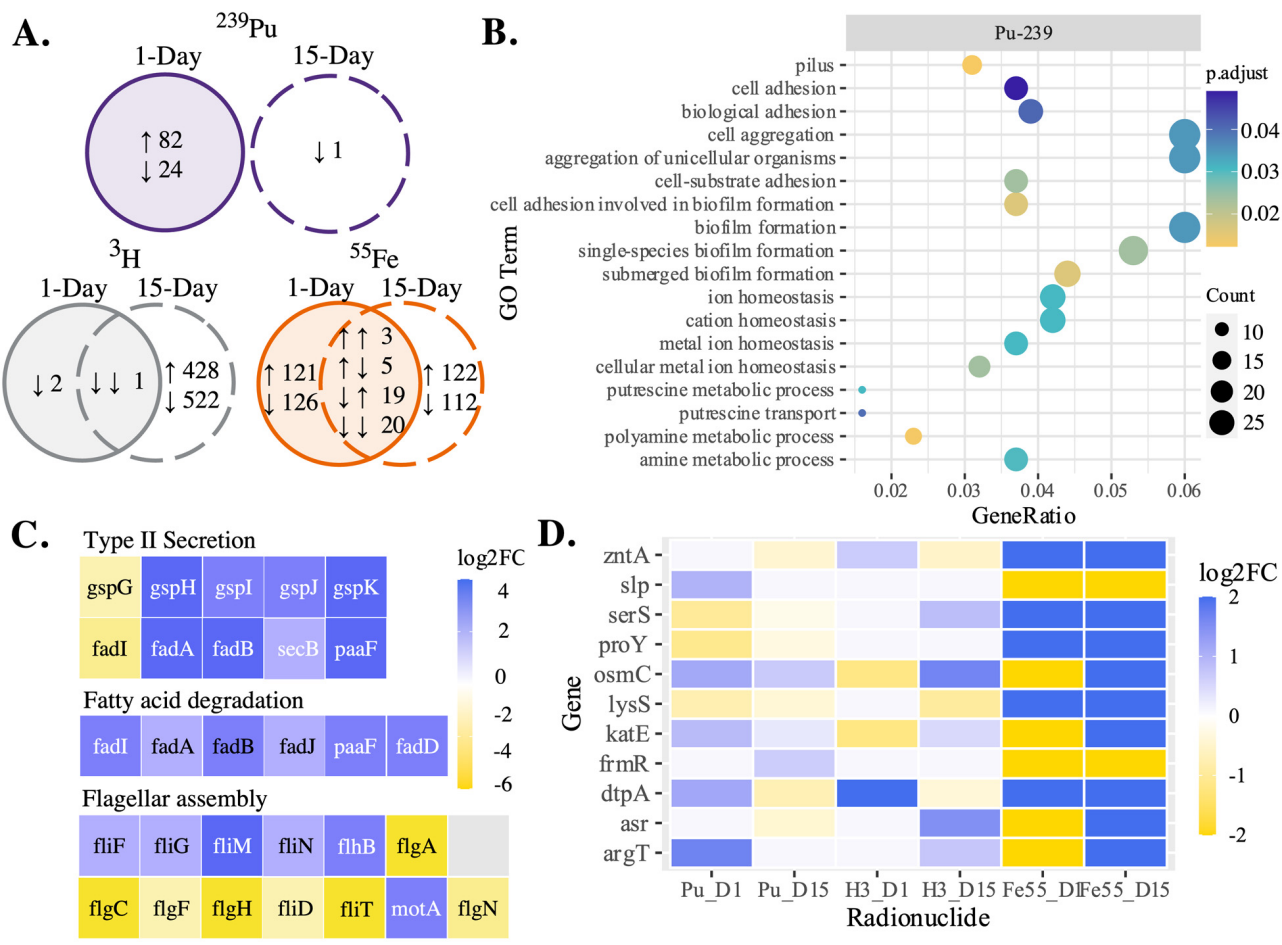
Unique response of *E. coli*. (A) Venn diagrams represent the number of differentially expressed genes in *E. coli* following 1 day and 15 days of exposure. (B) A dot plot displays GO terms uniquely enriched in *E. coli* following 1 day of ^239^Pu exposure as identified by overrepresentation analysis. The gene ratio represents the ratio of the number of significantly annotated genes over the total number in the term. (C) Select genes differentially expressed in *E. coli* by ^3^H exposure that encode components of T2SS, fatty acid degradation, and flagellar assembly. (D) A heat map displays select genes differentially expressed in *E. coli* following exposure to ^55^Fe, with color denoting the |log_2_ FC|.

Differential expression analysis identified genes in irradiated E. coli significantly changed from nonirradiated controls by a single radionuclide for the three different radionuclide sources considered (Fig. 7A). E. coli exposed to a ^239^Pu source revealed a moderate change in gene expression after 1 day of irradiation but negligible change after 15 days, with 106 genes (1.9% CDSs) and 1 gene (0.018% CDSs) uniquely differentially expressed, respectively (Fig. 7A, top). Responses induced by exposure to 1 day of ^239^Pu are broad with significant changes in gene expression of membrane proteins, fimbrial proteins, amino acid biosynthesis enzymes, and metal ion transporters. The gene encoding the regulatory protein RecA was downregulated by 1 day of exposure to ^239^Pu. Overrepresentation analysis revealed GO terms corresponding to genes involved in biofilm formation, cell adhesion and aggregation, metal ion homeostasis, and putrescine and polyamine metabolic processes were significantly changed by ^239^Pu exposure (Fig. 7B). After 15 days of exposure to ^239^Pu, only a single gene, *ynbA*, encoding CDP-alcohol phosphatidyltransferase domain-containing protein YnbA, was downregulated.

The change in gene expression in E. coli exposed to ^3^H was considerably higher after 15 days of continuous irradiation than after 1 day, with 950 (17.11% CDSs) and 2 (0.036% CDSs) uniquely differentially expressed genes, respectively (Fig. 7A). Only a single gene, *psiE*, was commonly differentially expressed at both times studied as represented by the union of the Venn diagram. The transcriptional response of E. coli induced by exposure to ^3^H for 15 days showed broad differential expression of polyketide biosynthesis components, the type II secretion system (T2SS), and fatty acid degradation (Fig. 7C). Flagellar assembly genes were also significantly changed in E. coli treated with ^3^H for 15 days, reflected by the upregulation of five genes and downregulation of 9 others.

Exposure of *E. coli* to ^55^Fe for 1 day and 15 days induced moderate change in gene expression, with 247 (4.4% CDSs) and 234 (4.2% CDSs) genes being uniquely differentially expressed (Fig. 7A). Unlike responses observed in E. coli exposed to ^239^Pu or ^3^H, exposure to ^55^Fe induced differential expression of 47 genes at both 1 day and 15 days. These functions of these genes were broad, with upregulation of genes encoding transporters and tRNA ligases for serine and lysine and downregulation of a gene encoding an osmotically inducible peroxiredoxin, ABC transporters, and DNA-binding transcriptional repressor (Fig. 7D). Stress response genes for acid shock, starvation, and peroxide stress were also differentially expressed in E. coli exposed to ^55^Fe for 1 day and 15 days.

## DISCUSSION

Understanding the biological response of environmental species to nuclear material is critical for the development of new detection methods and establishing proper dose-response relationships. Analysis of the transcriptional response of E. coli DH10β subjected *in situ* to exposure to ^239^Pu, ^3^H, and ^55^Fe for 1 day and 15-days provides a fundamental characterization of the effects of low-dose ionizing radiation on a model bacterium for the first time. E. coli was an ideal system to study the effects of low-dose ionizing radiation because it is well characterized (12, 16, 38) and previous work with radiation provides comparison with different sources and much higher dose rates (15, 21, 22, 25). Differential expression analysis revealed key similarities and differences in the gene expression of E. coli with three radionuclide sources and two times studied (Fig. 2 and 3).

The numbers of differentially expressed genes in E. coli varied among the three sources studied, despite similarities in average dose rates and exposure times. ^239^Pu exposure immediately induced stress on E. coli (Fig. 2A and B), ^3^H had a slower, delayed response (Fig. 2C and D), and ^55^Fe triggered similar levels of transcriptional activation at each time point studied (Fig. 2D and E). We surmise that the variation in transcriptional response is due to a combination of radiological (e.g., type or types of radiation emitted) and chemical (e.g., how the source is distributed within the culture) properties of the sources studied, although this requires further exploration. Prior studies have shown that ^239^Pu can bind to cell surfaces, interact with cells similarly to other heavy metals, and likely remain external to the cell without scavenging or addition of a chelator (39–42). The large initial transcriptional change is presumably due to major stress induced by the presence of ^239^Pu within the growth medium and possible sorption to the surface of the cells. The alpha particles emitted from ^239^Pu will deposit their energy densely, along a short path, suggesting that ^239^Pu sorbed to the surface of the cell could cause comparatively more stress than the other sources studied. In other words, the dose rate experienced locally (i.e., the microdosimetry) in cells from ^239^Pu is likely higher than the average dose rate across the entire culture. After the initial drastic response, E. coli appeared to adapt to the stress of ^239^Pu exposure and persisted in the 15-day data. Tritium can produce localized damage after incorporation into cells by substituting with hydrogen atoms and interacting with cellular material (43), although ^3^H will distribute uniformly in the culture without preference for which hydrogen it exchanges with. Tritium emits a low-energy beta particle, which will deposit energy much less densely than an alpha particle. We speculate the more homogeneous distribution of energy deposition at the average dose rate considered was not large enough to produce a measurable change after a single day of ^3^H exposure, but accumulation of these interactions over a longer period of time, like 15 days, was likely the cause of large-scale gene activation. Consistent changes in differential expression observed in E. coli treated with ^55^Fe compared to a stable iron counterpart over time indicate possible uptake and incorporation of iron via known binding sites and pathways (44). The resulting differential expression in E. coli reflects additional changes induced by ^55^Fe exposure over the stable control were constant over time.

Genes encoding components of carbohydrate metabolism (Table 1), the nuclear envelope, ABC transporters (Fig. 5), regulatory systems (see Fig. S4 in the supplemental material), siderophore biosynthesis (Table 3), and stress response pathways (Fig. 6) were differentially expressed in E. coli by more than one radionuclide. Several of these genes were upregulated by E. coli exposed to 15 days of ^3^H but downregulated by 1 day of exposure to ^239^Pu or ^55^Fe compared to corresponding nonirradiated E. coli. Common changes in gene expression with broad functions could indicate a general response to the dose rate studied (Fig. 3 and 4), and unique changes reveal radionuclide source-specific responses (Fig. 7).

Central carbohydrate metabolism is critical for cellular processes, and microorganisms adapt to environmental shifts and modified growth requirements by altering expression of metabolic genes (14). Differential expression analysis revealed expression changes in key carbohydrate metabolism genes in irradiated E. coli compared to nonirradiated E. coli (Fig. 4, Table 1, and Table 2). The upregulation of genes encoding gluconeogenesis enzymes in E. coli exposed to ^55^Fe or ^3^H for 15 days suggests the exposed samples have a higher biosynthetic burden, which requires gluconeogenesis under glucose-limited conditions (11, 45) (Table 1). The transition from exponential growth to stationary phase often results in the downregulation of TCA components, and downregulation of corresponding genes suggests ^55^Fe exposure could have heightened the response (46, 47) (Table 1). Oxidative phosphorylation is a metabolic pathway used by bacteria to acquire energy and during stationary phase influences survival (48, 49). Long-term ^3^H exposure induced upregulation of nearly all genes within the pathway, whereas short-term ^55^Fe exposure had an opposite response, indicating another route for energy production in ^3^H-treated E. coli (Table 1).

Limited nutrient availability in long-term stationary-phase cells can lead to amino acids serving as energy sources (50). Upregulation of several amino acid degradation pathways of lysine, valine, leucine, and isoleucine suggests possible breakdown of amino acids for energetic resources in E. coli exposed to ^3^H for 15 to days (Table 2). Moreover, similar exposure caused increased gene expression in E. coli of histidine biosynthesis genes, whose transcriptional regulation is triggered by amino acid starvation (51, 52). Long-term exposure to radiation in stationary phase possibly increased amino acid starvation in ^3^H-treated E. coli. Short-term ^239^Pu and ^55^Fe exposure downregulated these genes; however, 1-day cultures would not necessarily be under starvation conditions and require biosynthesis of histidine. Similar responses to increased amino acid starvation are not observed in long-term exposures to the other two radionuclide sources studied. It is possible that accumulation of ^55^Fe from long-term exposure induces alternative response mechanisms that are not reflective of amino acid starvation. Also, the lack of response in E. coli to ^239^Pu long-term exposure suggests possible adaptation to any induced stress or perceived stasis as the expression data indicate no significant changes in gene expression from the nonirradiated control levels.

The nuclear envelope plays a critical role in cellular homeostasis by responding to intrinsic and environmental stress in stationary-phase cells (53, 54). Previous studies have demonstrated the role of the nuclear envelope in combating stress caused by radiation exposure (55, 56). Upregulation of biosynthesis pathways of nuclear envelope components, peptidoglycan and lipopolysaccharides (LPSs), in addition to precursor molecules, O antigen and nucleotide sugars, by exposure to ^3^H in comparison to nonirradiated E. coli could indicate an adaptive response to radiation exposure to maintain homeostasis (Fig. S3). A previous study determining the effects of styrene on E. coli also found possible repair to the cell envelope through differential expression of similar nuclear envelope genes induced by ^3^H exposure (57). Differential expression analysis suggests biosynthesis of external and middle interior sections of the nuclear envelope but not the inner membrane in ^3^H-treated E. coli, with upregulation of glycerophospholipid metabolism genes and downregulation of fatty acid degradation genes after 15 days (Fig. 7C).

Enterobactin, a siderophore, is produced by E. coli to mediate iron transfer (58) and has been shown as a possible mechanism for the uptake of plutonium in E. coli (28). Exposure to 1 day of ^239^Pu induced upregulation of six enterobactin genes in E. coli (Table 3) required for synthesis, suggesting possible uptake of ^239^Pu by E. coli, which is consistent with observations in previous studies (28). These results may also suggest E. coli generated enterobactin to obtain other metals in order to combat initial stress induced by ^239^Pu exposure. In a similar study where E. coli was subjected to a different heavy metal, palladium, the same enterobactin genes were downregulated (59). Enterobactin synthesis genes were downregulated in ^3^H-treated E. coli after 15 days, indicating no measurable increase in gene expression compared to nonirradiated E. coli. Unsurprisingly, at either time analyzed, ^55^Fe exposure did not significantly change gene expression of enterobactin biosynthesis genes in E. coli as the control provided an equal amount of additional iron.

Bacteria have several strategies using signaling and cellular processes to control expression profiles in response to environmental changes and stress. The TCS is a predominant strategy consisting of histidine kinases and corresponding response regulators to sense and respond to environmental fluctuations by changing gene expression (60, 61), and in E. coli K-12, the TCS has been demonstrated to respond to nuclear envelope stress and metal sensing (62, 63). Osmotic stress, envelope stress, and high-zinc-level histidine kinase and cognate regulator genes were upregulated in E. coli following 15 days of ^3^H but were downregulated by 1 day of ^239^Pu or ^55^Fe exposure (Fig. S4). Increased transcription of TCSs could indicate a response by E. coli to combat stress induced by radiation as it would when subjected to osmotic and envelope stress (17), high concentrations of metals (63), or ethanol stress (62). Downregulation of a DNA-binding transcriptional activator and cognate histidine kinase pair, BasR and BasS, that responds to iron starvation (63) in ^55^Fe-treated E. coli, suggests repression of these genes. Secretion systems, including T2SS, play an important role in cellular communication (64) with numerous global functions of environmental adaptation, nutrients acquisition, and transport of folded proteins from the periplasm in Gram-negative bacteria (65, 66). Tritium exposure upregulated several general secretion pathway protein genes in E. coli after 15 days (Fig. 7C); however, general secretory or twin arginine targeting pathway genes that secrete proteins through the inner membrane were downregulated (67). These complex results suggest ^3^H exposure induces a transcriptional response of Gsp in E. coli but no measurable transcriptional increase of protein export genes.

The ATP binding cassette (ABC) transporter superfamily of proteins transports numerous nutrients and other molecules, such as heavy metal chelates, amino acids, and polysaccharides, across the membrane in E. coli (68, 69). Differential expression analysis of E. coli exposed to ^239^Pu, ^3^H, or ^55^Fe found significant changes in expression of genes coding for lipopolysaccharide (LPS), polyamine, amino acid, peptide, sugar, and metal ion ABC transporters (Fig. 4 and 5). The upregulation of genes involved in the export of external components, such as LPS, heme, siderophores, and lipoproteins, in ^3^H-treated E. coli aligns with increased gene expression of equivalent biosynthesis pathways. Putrescine and spermidine, common polyamines, and ABC transporters were upregulated in E. coli by exposure to 15 days of ^3^H and 1 day of ^239^Pu and downregulated by 15 days of exposure to ^55^Fe (Fig. 5 and Fig. 7B and C). Polyamines have several physiological functions in nucleic acid biosynthesis, response to oxidative stress, and cell growth (70) and are also used in response mechanisms to acid- and pH-induced stress. The transport of extracellular polyamines in response to radiation exposure could indicate a response to mediate fluctuations in environmental conditions or oxidative stress (71, 72). Moreover, upregulation of these components following 1 day of ^239^Pu exposure indicates the immediate stress of the presence of ^239^Pu on E. coli.

ABC transport importers of nutrients, such as sugars, amino acids, and ions, were also induced in irradiated E. coli (Fig. 5). The upregulation of genes encoding monosaccharide transporters for ribose, galactofuranose, and glycerol 3-phosphate in ^3^H-treated E. coli suggests transport of extracellular energy sources in a glucose-limited environment after 15 days of exposure. Tritium exposure induced upregulation of glutamine and histidine transporters in E. coli after 15 days. Upregulation of nitrogen cycling components such as nitrate reductase and glutamate synthase (Table 1) by 15 days of ^3^H exposure, but downregulation in short-term ^239^Pu and ^55^Fe exposures, indicates an underlying effect on nitrogen cycling to accommodate the stress of long-term low-dose radiation exposure. The trend is also observed in branched-chain amino acids (Table 2) that are involved in nitrogen regulation (73). Arginine transporters were upregulated in E. coli, indicating a response to stress induced by exposure to 1 day of ^239^Pu. A previous study found similar upregulation of arginine, histidine, and glutamine transporters in E. coli subjected to another heavy metal, palladium (59). Similarly, increased transcript levels of the metal, iron, and zinc transporters FecBCD, FhuBD, and ZnuABC (74), respectively, in irradiated E. coli compared to nonirradiated E. coli (Fig. 5) indicate a possible requirement for iron and zinc after 15 days. Downregulation of transporter genes in E. coli exposed to ^239^Pu or ^55^Fe could suggest minimal uptake of these molecules initially.

Another form of active transport, PTS, catalyzes both transport and phosphorylation via phosphoenolpyruvate (PEP) of various hexoses, such as glucose and amino sugars (75, 76), and has been demonstrated to be activated in E. coli during nutrient and oxidative stresses (13, 77). Downregulation of PTS genes (Fig. S4) suggests ^55^Fe-treated E. coli did not have a nutritional requirement for extracellular sugars compared to E. coli grown with an equimolar stable counterpart after initial exposure; however, upregulation of the same genes after 15 days of ^55^Fe and ^3^H exposure indicates a need to transport extracellular sugars in a glucose-limited environment. Additional sequencing of earlier time points in future studies may indicate if these genes were also differentially expressed in E. coli by ^239^Pu exposure between 1 day and 15 days that our data set did not capture.

Exposure to the radionuclides studied altered transcript levels of oxidative, osmotic, temperature, and general stress response genes in E. coli (Fig. 6); however, gene expression was not dominated by stress responses as observed at higher dose rates (1, 8). Oxidative stress is a common outcome of radiation exposure at higher doses and is induced following the production of reactive oxygen species through radiolysis (5, 15, 78, 79). The mechanisms to combat radiation-induced oxidative stress are well characterized in microorganisms (55, 80, 81). Genetic indicators of these mechanisms of a single superoxide dismutase (SOD) and a single catalase were initially downregulated in E. coli after short-term exposure to a ^3^H or ^55^Fe source but upregulated following long-term exposure (Fig. 6). These findings lack evidence to suggest overwhelming radiation induced superoxide or peroxide production since the majority of the genetic regulatory response to combat them, involving other SOD and catalase genes (82, 83), was not by genes significantly expressed in irradiated E. coli. Similar genes were also upregulated by E. coli subjected to stress induced by palladium, which suggests a correlation of response of long-term low-dose radiation exposure to heavy metal exposure (59). Upregulation of the transcriptional regulator OxyR and regulated genes (82, 84) following 15 days of ^3^H exposure (Fig. 6) could suggest possible increase and subsequent detoxification of peroxides in ^3^H-treated E. coli through OxyR regulation but no significant changes in ^239^Pu- or ^55^Fe-treated E. coli.

Induction of a global stress response regulated by RpoS, a sigma factor, permits cells to increase resistance to stress by regulating genes to repair damage or return to cellular homeostasis (38). Previous studies have demonstrated accumulation of RpoS E. coli in the stationary phase (85), upon stress or nutrient deprivation (16), or with oxidative stress induced by radiation exposure (21, 86). Differential expression analysis of irradiated E. coli revealed the levels of expression of several RpoS-mediated genes were significantly changed from those of their nonirradiated counterparts (Fig. 6). The initial downregulation of *rpoS* and an upstream outer membrane protein gene, *nlpD*, containing an internal promoter for the two genes (87) in E. coli by 1 day of ^3^H and ^55^Fe exposure and subsequent upregulation following 15 days of exposure indicate accumulation of the transcription factor and presence of the global stress response. There may be a collaborating system of signaling starvation conditions using the molecule (p)ppGpp, guanosine pentaphosphate, which upon increase also increases the transcription and translation of *rpoS* (88–90). Previous studies have also demonstrated the effects of (p)ppGpp on global transcription in E. coli during general and amino acid starvation (91, 92). Key genes in the biosynthesis of (p)ppGpp, *spoT*, and *gpp* (16, 93) were initially upregulated in E. coli following 1 day of ^55^Fe exposure (Fig. 6), suggesting the signaling pathway was initially induced by ^55^Fe.

Low-temperature- and high-osmolarity-induced stress can induce RpoS independent of (p)ppGpp regulation in stationary-phase E. coli (16, 20). Expression of the *otsAB* operon is vital for survival of stationary-phase E. coli in high osmolarity or following cold shock (19, 20). Upregulation of genes within the operon and an outer membrane protein A gene in E. coli following 15 days of ^3^H exposure and downregulation by a single day of ^239^Pu and ^55^Fe exposure (Fig. 6) suggest possible osmotic stress was induced alongside RpoS-mediated global stress response in E. coli by ^3^H and not by ^239^Pu or ^55^Fe. Genes encoding cold shock, chaperone, and heat shock proteins that were differentially expressed in E. coli by exposure to a ^239^Pu, ^55^Fe, or ^3^H source (Fig. 6) have also been shown to play a part in stress tolerance of bacteria and importance during cold and heat shock response (18, 94). Previous studies have shown induction of key heat shock proteins in bacteria subjected to gamma radiation (22, 95). Changes in the gene expression of these genes could indicate an adaptive response or tolerance to stress in irradiated E. coli compared to the nonirradiated counterpart (96).

DNA damage and repair are common in microorganisms following radiation exposure, especially with higher doses of ionizing radiation (3, 5, 25, 97). External stressors, such as styrene (57) and hydrogen peroxide (98), have been found to induce DNA damage in E. coli. Differential expression analysis provided some evidence of DNA strand break repair in E. coli, with moderate changes in genes with roles in repair pathways (Fig. 6). However, unlike with higher doses, there is minimal evidence for radiation-induced DNA strand breaks due to downregulation of repair genes in E. coli following 1 day of ^239^Pu or ^55^Fe exposure. Exposure to 15 days of ^3^H upregulated some repair genes; however, entire pathways used to repair different types of DNA breaks (3, 5) were not significantly overexpressed compared to nonirradiated E. coli.

In conclusion, the results and analysis presented in this study provide new insights to the transcriptional response of a model bacterium to *in situ* low-dose ionizing radiation exposure. The transcriptional response was broad, with significant changes in expression of genes encoding biosynthesis pathways of nuclear envelope components, amino acids, and siderophores, transport systems such as ABC transporters and type II secretion proteins, and initiation of stress response and regulatory systems of temperature stress, the RpoS regulon, and oxidative stress. The transcriptional profile of E. coli gained from this analysis expands the limited understanding of the effects of low-dose ionizing radiation on microorganisms and establishes fundamental science required for potential applications in bioremediation and development of biological sensing systems.

## MATERIALS AND METHODS

### Bacterial strain, media, and *in situ* exposure.

Escherichia coli DH10β (New England Biolabs) was cultivated in M9 minimal medium (99) supplemented with 0.5% glucose (Sigma-Aldrich), 1% thiamine hydrochloride (Spectrum), and 1% (wt/vol) Casamino Acids (Bacto) at 28°C and with shaking at 215 rpm in the presence or absence of a radionuclide source for the duration of the 15-day period of this study. Components were added in this order: (i) 100 mL of M9, (ii) the radionuclide source, and (iii) overnight E. coli culture (see Fig. S1A in the supplemental material). Radionuclides in solution were added once at the beginning of the period of study to 100 mL of M9 in 250-mL baffled flasks to achieve final activity concentrations that would yield an approximate absorbed dose rate of 10 mGy day^−1^. Target radionuclide medium concentrations were calculated based on simplified approximations of the absorbed dose rate, assuming homogeneous, consistent distribution of the radionuclides in the medium. That is, the absorbed dose rate was calculated as the rate of energy of the major particle(s) emitted (National Nuclear Data Center) from the radionuclides relative to the mass of the medium (32). There is acknowledged uncertainty here as different radionuclides will distribute differently within the culture over the duration of the experiment, and this approximation does not account for particles that escape the medium (i.e., “edge effects” where a particle deposits its energy in the glass of the flask, for example, rather than in the culture), but we consider this a reasonable point of reference to initiate comparisons. Note that the dose rate from ^55^Fe was calculated based on electron emissions.

^239^Pu in 0.01 mM HCl in-house stock was added to culture flasks to achieve a concentration of ~50 ppb (~0.12 kBq mL^−1^). Tritium as tritiated water (HTO) (Perkin Elmer) was added to achieve a final activity concertation of ~0.3 ppb (~0.11 MBq mL^−1^). Finally, ^55^FeCl_3_ in 0.5 M HCl (Perkin Elmer) was added for a final activity concentration of ~2 ppb (~0.18 MBq mL^−1^).

Single colonies of E. coli cells were grown overnight in 3 mL of M9. Following radionuclide addition to the growth medium, overnight cultures, adjusted to an OD_600_ of 0.1, were added to each sample flask. Nonirradiated control groups were prepared as described above but without radionuclide source addition. Stable 2.47 μM FeCl_3_ in 0.5 M HCl control was utilized (2 ppb) for comparison with the radioactive iron counterpart. Although plutonium does not have a stable isotope, the low mass concentration is not anticipated to result in chemically induced effects. Independent biological triplicate flasks were used for the radionuclide and control groups. Growth of E. coli was monitored with OD_600_ measurements throughout the 15-day study (Fig. S1B). Cultures were incubated for sampling at 28°C and shaking at 215 rpm for 15 days, and samples for downstream sequencing analysis were removed at 1 day and 15 days.

### Total RNA isolation and rRNA depletion.

E. coli cultures grown with and without radionuclide source addition were harvested after 1 day and 15 days and normalized to an OD_600_ of 1.0 to obtain a cell density of 5.0 × 10^7^ CFU mL^−1^. Cells were stabilized by incubation with 1 mL of RNAprotect bacterial reagent (Qiagen) for 10 min at room temperature. Following incubation, samples were centrifuged at 5,500 × *g* for 6 min at room temperature, and the supernatant was decanted. Pellets were flash-frozen with liquid nitrogen and stored at –80°C.

Total RNA was extracted from biological triplicates of 1-day and 15-day irradiated and nonirradiated samples according to the manufacturer’s instructions of the RNeasy Protect bacterial minikit (Qiagen) with an on-column DNase I digestion for 20 min. Cell pellets were lysed in 200 μL 5 mg mL^−1^ lysozyme (Sigma) in 10 mM Tris-hydrochloride (Sigma)–1 mM EDTA (Becton Dickinson) (pH 8.0) at room temperature for 10 min. RNA was eluted in 35 mL of molecular-grade water, flash-frozen in liquid nitrogen, and stored at –80°C. If the concentration was low, the eluent was reapplied to the spin column, incubated for 10 min at room temperature, and reprocessed. The total RNA concentration, purity, and quality were quantified on a Qubit 4 fluorometer (Invitrogen), Bioanalyzer 2100 (Agilent Technologies), and NanoDrop 2000 spectrophotometer (Thermo Scientific), respectively.

rRNA was depleted from ^239^Pu- and ^55^Fe-irradiated samples as well as nonirradiated control samples using the Ribo-Zero rRNA removal kit for bacteria (Illumina) according to the manufacturer’s instructions. Tritium and stable FeCl_3_ samples were depleted of rRNA using the manufacturer’s instructions for the Ribominus Bacteria 2.0 transcriptome isolation kit (Invitrogen), followed by ethanol precipitation. Two different kits were used as Ribo-Zero was discontinued by the manufacturer before we completed our study. Quality control of depleted samples followed the same protocols as described for total RNA.

### Library preparation and RNA sequencing.

The TruSeq stranded mRNA library prep kit was used for cDNA library preparation following the low-sample LS protocol as previously described (100). Library concentration, quality, and size were verified through KAPA (Roche) validation, Qubit (Invitrogen), and a high-sensitivity DNA chip (Agilent Technologies) on an Agilent Bioanalyzer 2100 before and after sample pooling. Libraries were pooled in equimolar amounts. Samples were sequenced on a NextSeq550 high-output v.2 platform (Illumina) with 300 cycles for 2 × 150-bp paired-end reads.

### Bioinformatic analysis of RNA-seq data.

Raw strand-specific reads were demultiplexed and quality checked, and adapters were trimmed by Trim Galore using Cutadapt (v.2.8) commands (101, 102). Computational resources were provided by the Palmetto Cluster. Reads were checked for quality using fastQC (v.0.11.8) (103). Paired-end reads were aligned to the RefSeq annotation of E. coli K-12 MG1655 (RefSeq GCF_000005845.2) with HISAT2 (v.2.2.1) (104). Transcripts were assembled using Stringtie (v.1.3.3) with genome-guided mapping following SAMtools (v.1.4) conversion (105–107). Count tables were generated with tximport (v.1.20.0) using Stringtie output. DESeq2 (v.1.35.0) was used to determine differential expression from each data set (108). Parameters of log_2_ fold change from nontreated controls of >2 and an adjusted Wald test *P* value of <0.05 were used as criteria for differential expression.

Filtered data sets were analyzed to find genes whose expression was differentially expressed by only a single isotope using jVenn software (109). Differentially expressed genes from all data sets were compared to identify unique genes. Gene identifiers and log_2_ fold changes for unique data sets were then input into an R script of UNC’s Pathview pathway mapper (110) for mapping of genes to E. coli pathways against Kyoto Encyclopedia of Genes and Genomes (KEGG) annotation *eco* (36, 111). Commands from clusterProfiler (v.4.0.2) were used to perform overrepresentation analysis of differential expression data with enrichGO for Gene Ontology (GO) and enrichKEGG for KEGG pathways (34, 35).

### Data availability.

RNA-seq data sets for this study have been deposited in the GEO database under accession no. GSE208658.
