# Erratum for Wintenberg et al., “Global Transcriptional Response of *Escherichia coli* Exposed *In Situ* to Different Low-Dose Ionizing Radiation Sources”

**DOI:** 10.1128/msystems.00159-25

**Published:** 2025-02-12

**Authors:** Molly Wintenberg, Lisa Manglass, Nicole E. Martinez, Mark Blenner

## ERRATUM

Volume 8, no. 2, e00718-22, 2023, https://doi.org/10.1128/msystems.00718-22. Page 18, Acknowledgments, second sentence: “P20GM139767” should read “P20GM139769.”

