# Supplementary material for: Global Transcriptional Response of Escherichia coli Exposed In Situ to Different Low-Dose Ionizing Radiation Sources
Source: mSystems. 2023 Feb 13;8(2):e00718-22. doi: 10.1128/msystems.00718-22 (PMC10134817; doi:10.1128/msystems.00718-22)
Supplement: FIG S1 [file msystems.00718-22-s0001.pdf]

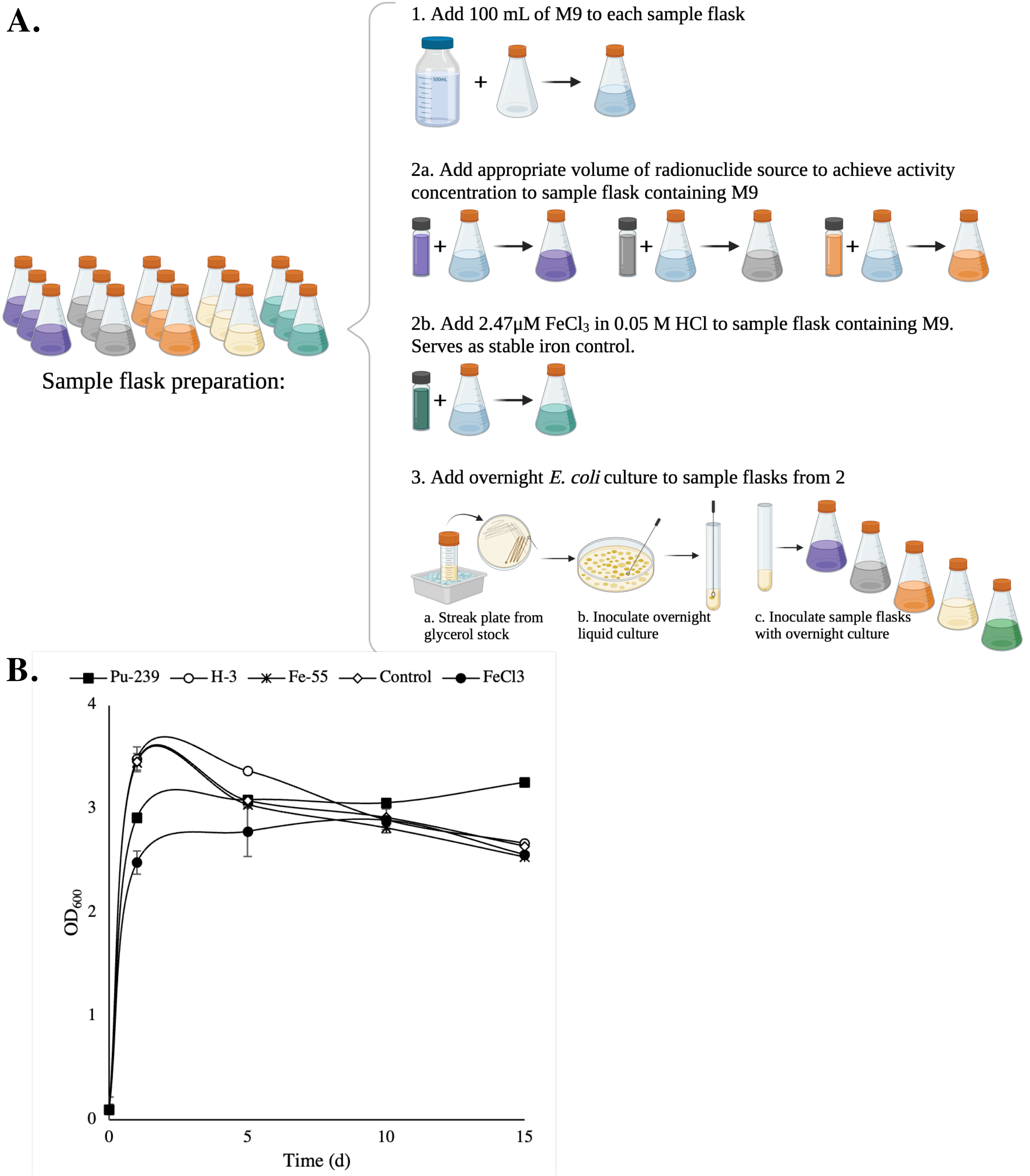
A.
Sample flask preparation:
1. Add 100 mL of M9 to each sample flask
500mL
2a. Add appropriate volume of radionuclide source to achieve activity concentration to sample flask containing M9
2b. Add 2.47μM FeCl3 in 0.05 M HCl to sample flask containing M9. Serves as stable iron control.
3. Add overnight E. coli culture to sample flasks from 2
a. Streak plate from glycerol stock
b. Inoculate overnight liquid culture
c. Inoculate sample flasks with overnight culture
B.
Pu-239
H-3
Fe-55
Control
FeCl3
OD600
Time (d)
0
1
2
3
4
0
5
10
15
