# Supplementary figures and images for: Global Transcriptional Response of Escherichia coli Exposed In Situ to Different Low-Dose Ionizing Radiation Sources
Source: mSystems. 2023 Feb 13;8(2):e00718-22. doi: 10.1128/msystems.00718-22 (PMC10134817; doi:10.1128/msystems.00718-22)

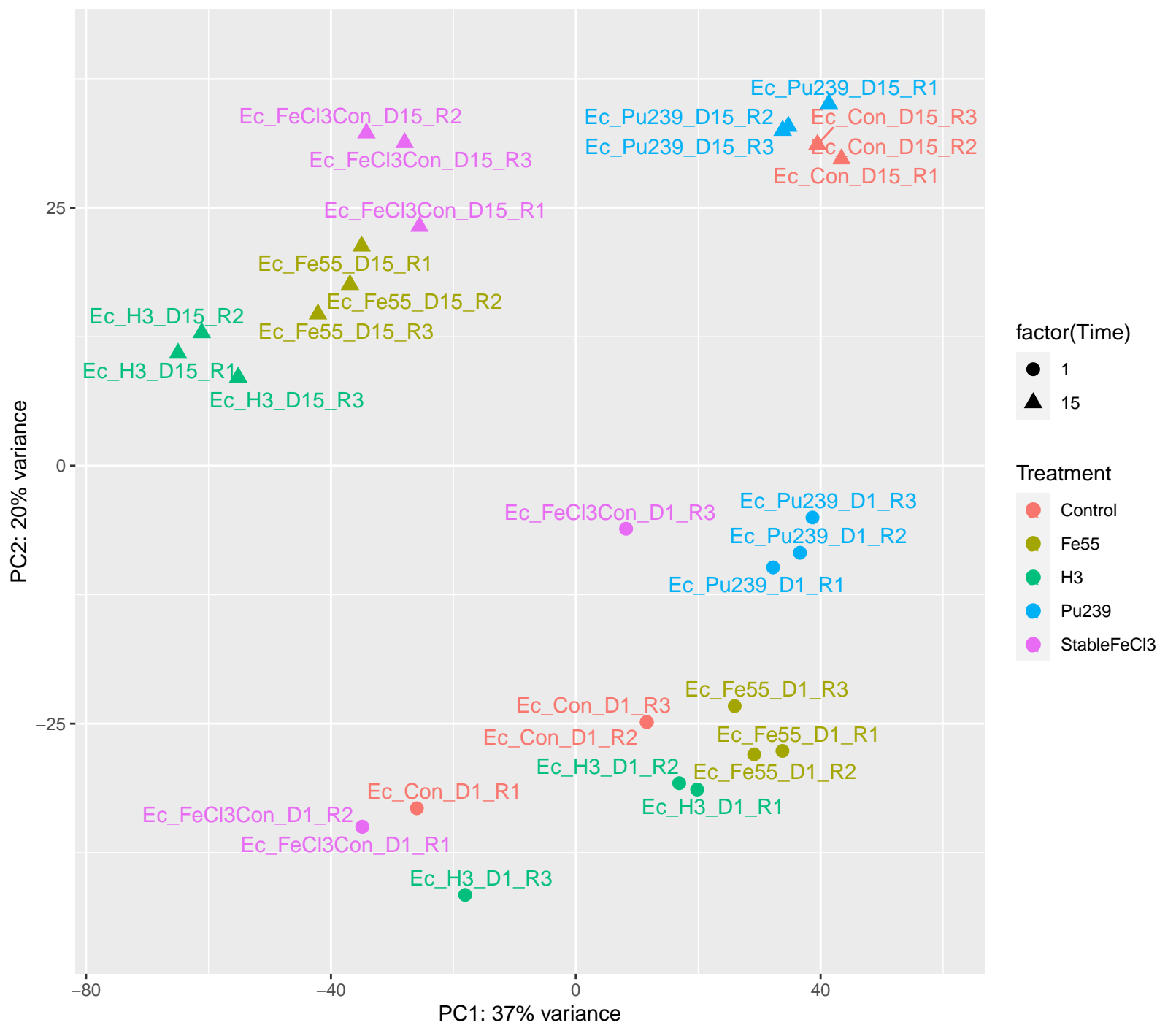

Supplement: FIG S2 [file msystems.00718-22-s0002.pdf]

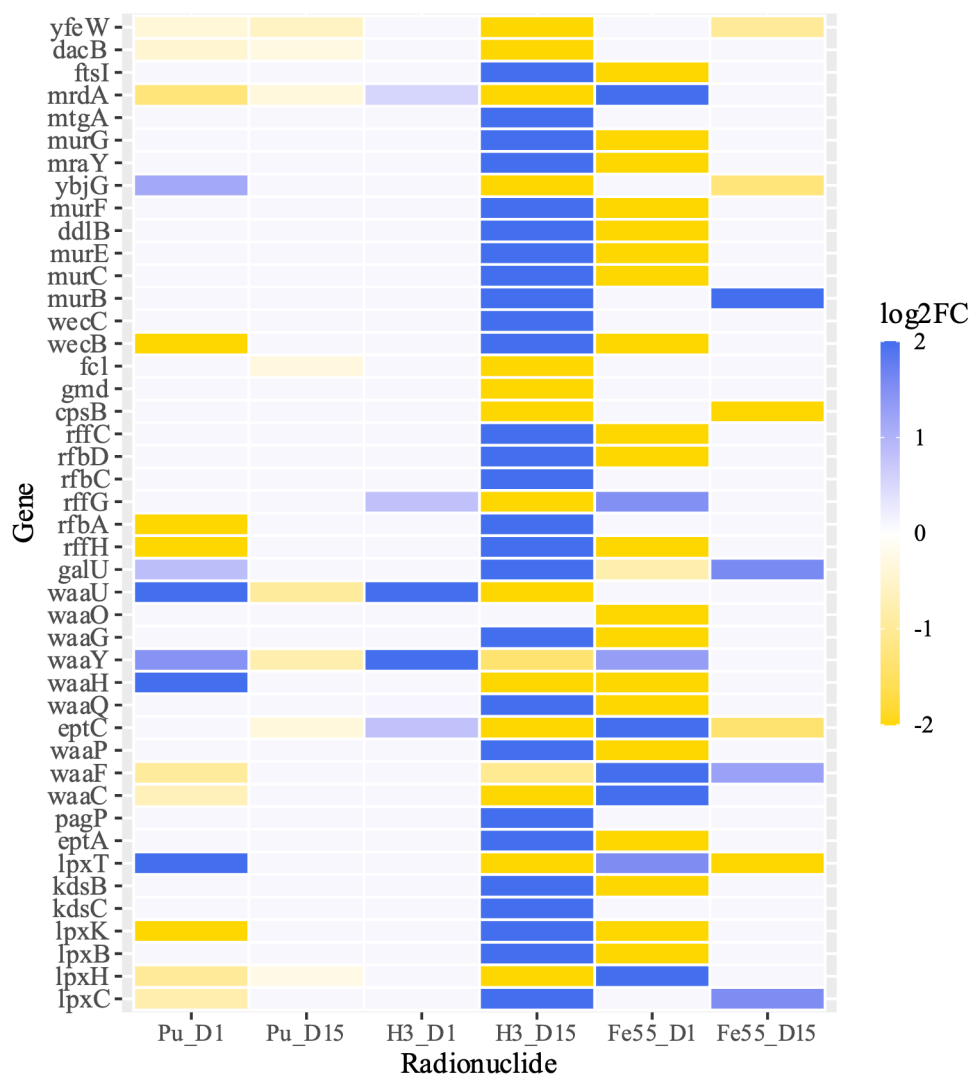

Supplement: FIG S3 [file msystems.00718-22-s0003.pdf]

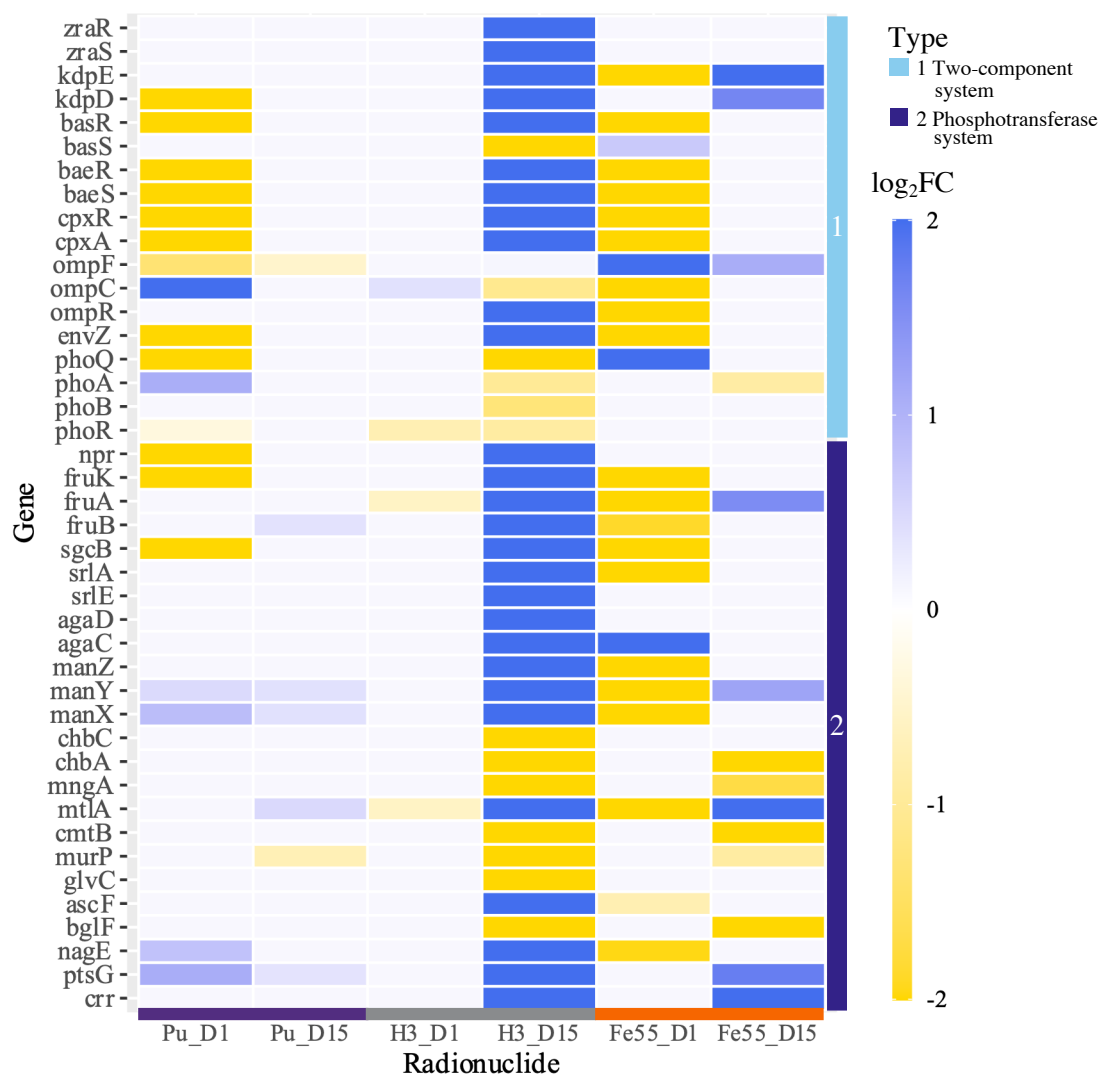

Supplement: FIG S4 [file msystems.00718-22-s0004.pdf]
