# Supplementary material for: Global Transcriptional Response of Escherichia coli Exposed In Situ to Different Low-Dose Ionizing Radiation Sources
Source: mSystems. 2023 Feb 13;8(2):e00718-22. doi: 10.1128/msystems.00718-22 (PMC10134817; doi:10.1128/msystems.00718-22)
Supplement: TABLE S1 [file msystems.00718-22-s0005.docx]

**Table S1.** Characteristics of RNA-seq data in this study.

| **Sample ID** | **Type** | **Time (d)** | **Rep** | **Total Reads** | **Reads for Alignment** | **Overall Align Rate (%)** | **Reads Aligned** | **GEO Accession No.** |
| --- | --- | --- | --- | --- | --- | --- | --- | --- |
| Ec_Con_D1_R1 | Control | 1 | 1 | 14,325,164 | 14,310,935 | 93.4 | 13,367,844 | GSM6360726 |
| Ec_Con_D1_R2 | Control | 1 | 3 | 26,749,366 | 26,643,570 | 94.1 | 25,082,257 | GSM6360727 |
| Ec_Con_D1_R3 | Control | 1 | 3 | 14,233,732 | 14,166,664 | 94.2 | 13,342,164 | GSM6360728 |
| Ec_Con_D15_R1 | Control | 15 | 1 | 19,770,602 | 19,280,098 | 91.6 | 17,656,714 | GSM6360729 |
| Ec_Con_D15_R2 | Control | 15 | 2 | 28,944,111 | 27,770,835 | 88.3 | 24,510,539 | GSM6360730 |
| Ec_Con_D15_R3 | Control | 15 | 3 | 14,416,116 | 13,821,235 | 87.7 | 12,121,233 | GSM6360731 |
| Ec_Pu239_D1_R1 | Pu-239 | 1 | 1 | 16,356,011 | 15,903,587 | 90.5 | 14,389,566 | GSM6360732 |
| Ec_Pu239_D1_R2 | Pu-239 | 1 | 3 | 15,383,341 | 14,727,663 | 89.0 | 13,104,675 | GSM6360733 |
| Ec_Pu239_D1_R3 | Pu-239 | 1 | 3 | 32,190,571 | 31,338,129 | 91.1 | 28,549,036 | GSM6360734 |
| Ec_Pu239_D15_R1 | Pu-239 | 15 | 1 | 16,199,569 | 15,638,496 | 88.9 | 13,907,314 | GSM6360735 |
| Ec_Pu239_D15_R2 | Pu-239 | 15 | 2 | 15,618,853 | 15,067,139 | 89.1 | 13,430,848 | GSM6360736 |
| Ec_Pu239_D15_R3 | Pu-239 | 15 | 3 | 15,102,984 | 14,640,428 | 90.1 | 13,188,098 | GSM6360737 |
| Ec_H3_D1_R1 | HTO | 1 | 1 | 21,952,366 | 21,805,392 | 92.8 | 20,241,945 | GSM6360738 |
| Ec_H3_D1_R2 | HTO | 1 | 3 | 4,878,183 | 4,869,705 | 92.1 | 4,483,537 | GSM6360739 |
| Ec_H3_D1_R3 | HTO | 1 | 3 | 15,791,521 | 15,760,095 | 93.0 | 14,660,040 | GSM6360740 |
| Ec_H3_D15_R1 | HTO | 15 | 1 | 12,071,136 | 12,016,267 | 94.1 | 11,303,702 | GSM6360741 |
| Ec_H3_D15_R2 | HTO | 15 | 2 | 20,371,224 | 20,288,701 | 93.2 | 18,905,012 | GSM6360742 |
| Ec_H3_D15_R3 | HTO | 15 | 3 | 27,691,163 | 27,630,010 | 94.4 | 26,082,729 | GSM6360743 |
| Ec_Fe55_D1_R1 | Fe-55 | 1 | 1 | 25,650,585 | 24,998,657 | 95.7 | 23,926,215 | GSM6360744 |
| Ec_Fe55_D1_R2 | Fe-55 | 1 | 3 | 14,533,530 | 14,126,181 | 93.9 | 13,261,659 | GSM6360745 |
| Ec_Fe55_D1_R3 | Fe-55 | 1 | 3 | 14,217,168 | 13,984,098 | 96.2 | 13,488,507 | GSM6360746 |
| Ec_Fe55_D15_R1 | Fe-55 | 15 | 1 | 10,606,224 | 10,342,147 | 93.5 | 9,665,771 | GSM6360747 |
| Ec_Fe55_D15_R2 | Fe-55 | 15 | 2 | 8,217,403 | 7,956,240 | 91.5 | 7,278,368 | GSM6360748 |
| Ec_Fe55_D15_R3 | Fe-55 | 15 | 3 | 9,755,229 | 9,573,268 | 94.6 | 9,057,269 | GSM6360749 |
| Ec_FeCl3Con_D1_R1 | FeCl3 | 1 | 1 | 2,264,074 | 2,252,114 | 71.6 | 1,612,514 | GSM6360750 |
| Ec_FeCl3Con_D1_R2 | FeCl3 | 1 | 3 | 4,259,040 | 4,238,313 | 93.3 | 3,954,770 | GSM6360751 |
| Ec_FeCl3Con_D1_R3 | FeCl3 | 1 | 3 | 9,842,573 | 9,830,919 | 95.7 | 9,403,274 | GSM6360752 |
| Ec_FeCl3Con_D15_R1 | FeCl3 | 15 | 1 | 35,281,286 | 35,107,667 | 32.1 | 11,262,540 | GSM6360753 |
| Ec_FeCl3Con_D15_R2 | FeCl3 | 15 | 2 | 14,187,718 | 14,143,805 | 44.9 | 6,350,568 | GSM6360754 |
| Ec_FeCl3Con_D15_R3 | FeCl3 | 15 | 3 | 27,159,521 | 27,087,376 | 42.0 | 11,376,698 | GSM6360755 |
